# Bioactive self-healing hydrogel based on tannic acid modified gold nano-crosslinker as an injectable brain implant for treating Parkinson’s disease

**DOI:** 10.1186/s40824-023-00347-0

**Published:** 2023-02-08

**Authors:** Junpeng Xu, Tsai-Yu Chen, Chun-Hwei Tai, Shan-hui Hsu

**Affiliations:** 1 Institute of Polymer Science and Engineering, National Taiwan University, No. 1, Sec. 4 Roosevelt Road, Taipei, 10617 Taiwan, Republic of China; 2 Department of Neurology, National Taiwan University Hospital, No.7, Zhongshan South Road, Zhongzheng District, Taipei, 100225 Taiwan, Republic of China; 3 Institute of Cellular and System Medicine, National Health Research Institutes, No. 35 Keyan Road, Miaoli, 35053 Taiwan, Republic of China

**Keywords:** Bioactive hydrogel, Self-healing, Parkinson’s disease, Tannic acid, Gold nanoparticle, Conductive hydrogel

## Abstract

**Background:**

Parkinson’s disease (PD) is one of the most common long-term neurodegenerative diseases. Current treatments for PD are mostly based on surgery and medication because of the limitation and challenges in selecting proper biomaterials. In this study, an injectable bioactive hydrogel based on novel tannic acid crosslinker was developed to treat PD.

**Methods:**

The oxidized tannic acid modified gold nano-crosslinker was synthesized and used to effectively crosslink chitosan for preparation of the bioactive self-healing hydrogel. The crosslinking density, conductivity, self-healing ability, and injectability of the hydrogel were characterized. Abilities of the hydrogel to promote the proliferation and differentiation of neural stem cells (NSCs) were assessed in vitro. Anti-inflammatory property was analyzed on J774A.1 macrophages. The hydrogel was injected in the PD rat model for evaluation of the motor function recovery, electrophysiological performance improvement, and histological repair.

**Results:**

The hydrogel exhibited self-healing property and 34G (~ 80 μm) needle injectability. NSCs grown in the hydrogel displayed long-term proliferation and differentiation toward neurons in vitro. Besides, the hydrogel owned strong anti-inflammatory and antioxidative capabilities to rescue inflamed NSCs (~ 90%). Brain injection of the bioactive hydrogel recovered the motor function of PD rats. Electrophysiological measurements showed evident alleviation of irregular discharge of nerve cells in the subthalamic nucleus of PD rats administered with the hydrogel. Histological examination confirmed that the hydrogel alone significantly increased the density of tyrosine hydroxylase positive neurons and fibers as well as reduced inflammation, with a high efficacy similar to drug-loaded hydrogel.

**Conclusion:**

The new bioactive hydrogel serves as an effective brain injectable implant to treat PD and a promising biomaterial for developing novel strategies to treat brain diseases.

**Graphical Abstract:**

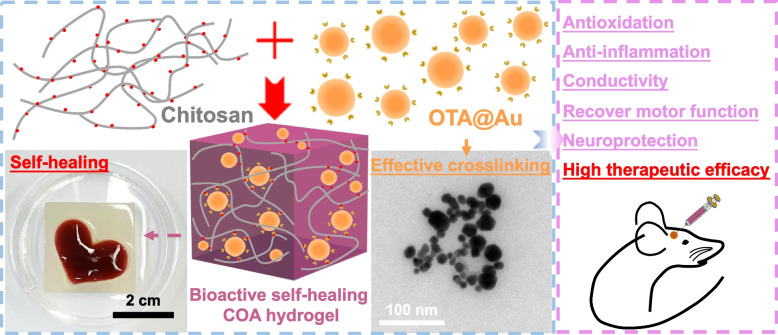

**Supplementary Information:**

The online version contains supplementary material available at 10.1186/s40824-023-00347-0.

## Introduction

Parkinson’s disease (PD) is a common age-related neurodegenerative disease with progression and incurability [1]. The pathological feature of PD is the region-specific loss of nigrostriatal dopaminergic neurons and of their projected nerve fibers to the striatum [2]. The deficiency of dopaminergic neurons in the substantia nigra pars compacta (SNpc) causes the irregular discharge of subthalamic nucleus (STN), resulting in serious behavioral symptoms such as tremor and bradykinesia to affect the daily routine of patients [3, 4]. Meanwhile, dopaminergic neurons in SNpc are particularly susceptible to oxidative stress due to their operation in hyperoxidative conditions, which is also associated with the low antioxidant glutathione level and iron content [5, 6]. Oxidative stress stimulates feedforward loops of the inflammation and death of neuronal cells leading to microglia generation of reactive oxygen species (ROS), which in turn leads to the death of dopaminergic neurons that ultimately propagates and drives the disease progression [7]. While there is no cure method for PD currently, the clinical treatment such as surgery or drug therapy can help relieve symptoms. The treatment by surgery may have unsatisfactory or side effects and the drug therapy may be affected by blood-brain barrier, reducing the efficiency of treatment [8]. Therefore, several new-generation therapeutic strategies for PD have been proposed based on the use of biomaterials especially hydrogels carrying various bioactive factors [9].

Hydrogels, with highly hydrophilic three-dimensional network polymeric networks, are crosslinked through intermolecular or intramolecular interaction [10]. Self-healing hydrogels which are crosslinked via dynamic bonding (e.g., Schiff base linkage) attract special interests because they mimic the healing mechanisms existing in living organisms [11]. Biocompatible self-healing hydrogels are often prepared from natural or derived polymers including chitosan and tannins [12]. Among these polymers, chitosan is a renewable polysaccharide with biocompatibility, biodegradability, and antibacterial property and has been widely employed in the biomedical field. The major limitation of chitosan for biomedical applications is its poor solubility in neutral water [13]. Carboxymethyl modification of chitosan improves the solubility and reactivity of chitosan without affecting the biocompatibility, thus making the carboxymethyl chitosan (CMC) an excellent candidate for preparation of hydrogels [14]. Meanwhile, tannic acid (TA) is a natural high phenolic hydroxyl molecule, and has excellent ROS scavenging ability and biocompatibility as well as possibility for functionalization [15]. Recent studies have shown that tannins as bioactive components have neuroprotective effects on neurodegenerative diseases through multiple actions including anti-inflammatory activities, antioxidant capacity, modulation of cell signaling pathways, and anti-amyloid actions [16, 17]. These unique features make TA a promising ingredient to prepare biomaterials to treat neural degenerative diseases or brain injury. Although TA has been used as a component of hydrogel for wound dressings [18], the incorporation of TA into a polymeric network for synthesizing an injectable hydrogel to treat brain diseases or injury is not explored.

Conductive hydrogels are a fascinating category of hydrogels for the repair and regeneration of electroactive tissues, particularly neural tissues [19]. Various electroactive materials can be employed as ingredients or additives in hydrogels to bring conductivity, such as conductive polymers and metal nanoparticles [20], but they rarely possess bioactivity. For instance, a chitosan-based hydrogel with conductive gold nanoparticles (AuNPs) has been developed to be thermo-sensitive for cardiac tissue engineering [21]. A few studies showed that AuNPs were effective in treating PD, probably because AuNPs presented antioxidant and neuroprotective effects on brain cells [22, 23]. Very recently, colloid AuNPs were added in a chitosan-polyurethane self-healing hydrogel, which through animal studies unveiled the potential of using conductive hydrogel alone in treating PD [24]. This work suggested that conductive hydrogels may not only be used as a drug/cell carrier, but also as curative tissue engineering scaffold materials. However, the efficacy of conductive hydrogel alone has not been compared with a curative agent in PD studies.

Nanoparticles made from the combination of AuNPs and TA were explored as an additive to stimulate the dendritic cell activation in literature [25]. Such composite nanoparticles do not present any quinone groups and cannot serve as a Schiff crosslinker. Meanwhile, TA can be effectively functionalized with quinone using the enzyme laccase through oxidized self-polymerization, but such an oxidized tannic acid (OTA) did not provide sufficient crosslinking and required the presence of potentially toxic Fe^3+^ for double crosslinking to strengthen the network [26]. In the current study, a new and more effective nano-crosslinker, i.e., OTA modified AuNPs (OTA@Au), was successfully developed and used to prepare an injectable bioactive self-healing hydrogel platform via dynamic Schiff reaction with chitosan. The promotive effect of this bioactive self-healing hydrogel on the proliferation and differentiation of NSCs was examined. The potential therapeutic function of the hydrogel in PD treatment was evaluated by antioxidative and anti-inflammatory assays in vitro as well as the injection of the hydrogel in the brain of PD-induced rats in vivo. Besides, the efficacy of the bioactive hydrogel and that of the drug-loaded hydrogel were compared to verify the functionality and potential of using the bioactive hydrogel alone as the brain implant for treating PD. The new hydrogel also presents the potential as an implant to suppress the neuroinflammation and protect the neural cell function.

## Materials and methods

### Synthesis of oxidized tannic acid (OTA) and OTA modified gold nanoparticles (OTA@Au)

Tannic acid (TA, ACS reagent, Sigma-Aldrich, USA) was oxidized by laccase (from Trametes versicolor, 0.9 U/mg, Sigma-Aldrich, USA) to yield oxidized tannic acid (OTA) according to previous literature with modification [26]. TA (0.425 g) were well dissolved in a 25 mL, phosphate buffer (pH ~ 6.5) at 30 °C, and then added with 0.111 g (100 U) of laccase under constant stirring at 800 rpm for 24 h to induce the oxidative self-polymerization of TA. The reaction product was OTA. The OTA suspension was centrifuged at 8000 rpm for 1 h and rinsed three times with DI water (resistivity = 18.2 MΩ/cm). The sediment was collected and then lyophilized for 48 h to obtain the OTA powder for further synthesis.

The synthetic method of OTA modified gold nanoparticles (OTA@Au) was designed based on an improvement on the method of producing TA modified Au in the previous work [25]. OTA@Au with a weight concentration of Au equal to 100 ppm in colloidal suspension was prepared in DI water by reduction of gold (III) chloride trihydrate (Sigma-Aldrich, ≥99.9% trace metals basis). The aqueous solution of gold (III) chloride trihydrate (0.018 wt%) was boiled and vigorously stirred under reflux. Then, the aqueous solution of OTA (5 wt%) was added into the boiling solution. After the reduction mixture (50 g) turned red in color, i.e., gold nanoparticles (AuNPs) formed, the colloid was further stirred at reflux for another 15 min and cooled to room temperature. The pH value of the obtained OTA@Au colloid solution was adjusted to neutral by adding NaOH solution.

### Characterization of OTA and OTA@Au

The successful synthesis of OTA was validated by liquid Fourier transform infrared spectroscopy (IR, Spectrum 100, PerkinElmer) with a wavenumber range from 4000 to 1000 cm^− 1^. The carbonyl content of OTA was investigated by the titrimetry [27]. OTA powder (0.1 g) was suspended in 10 mL of DI water with heating to 40 °C followed by adjusting the pH value to ~ 3.2 using HCl. The hydroxylamine reagent (1.6 mL) was added and stirred for 4 h. Determination of excess hydroxylamine was performed by rapid titration of the reaction mixture with 0.1 M HCl to a pH of 3.2. The hydroxylamine reagent was prepared by dissolving hydroxylamine hydrochloride (Fluka, Japan) in NaOH solution with the concentration of 5 wt%. The carbonyl content was calculated with Eq. (1):

1 $$percentage\ of\ carbonyl\ content=\frac{\left[\left({V}_0-V\right)\times M\times 0.028\right]}{W}\times 100\%$$

where *V*_0_ is the volume of HCl used for the blank (mL), *V* is the volume of HCl required for the sample (mL), *M* represents the molarity of HCl, and *W* represents the sample weight (g).

The morphological images were obtained by transmission electron microscopy (TEM, Hitachi H-7100, Japan). Ultraviolet-visible (UV–Vis) spectroscopy was employed to verify the presence of the characteristic peaks of AuNPs and carbonyl groups in OTA@Au. A UV-Vis spectrometer (SpectraMax M5, Molecular Devices, USA) was used to scan each solution in the wavelength range of 200 nm to 800 nm. The presence of the carbonyl group is determined by the characteristic absorbance peak at 360 nm and the formation of AuNPs is judged by the emergence of the absorption peak at ~ 524 nm. The ζ-potentials and averaged hydrodynamic diameters were calculated by a nanoparticle analyzer (Delsa Nano, Beckman Counter). The carbonyl content of the OTA@Au nano-crosslinker was also estimated by the former mentioned titrimetric method.

### Preparation of O-carboxymethyl chitosan (CMC)/OTA@Au (COA) hydrogels

The composite hydrogel, i.e., COA hydrogels, were prepared by mixing O-carboxymethyl chitosan (CMC, Mw ∼ 300,000, Biosynth-carbosynth, UK) and OTA@Au. Higher contents of OTA@Au used in this paper (300 ppm, 400 ppm, and 500 ppm) were obtained by concentration under reduced pressure. Then, the concentrated OTA@Au solution was added into CMC aqueous solution (5 wt%) under vortex at 25 °C to obtain a homogeneous precursor, resulting in the formation of COA hydrogel with simultaneous crosslinking.

### Physico-chemical and rheological properties of COA hydrogels

The conventional four-probe method was used to measure the conductivity of each hydrogel using a single-channel system source meter instrument (2601B, Keithley) under 0.5 V at 25 °C. The porosity was assessed by the ethanol soaking method. The porosity value was implemented by the Eq. (2):

2 $$porosity=\frac{W_s-{W}_d}{\rho V}\times 100\%$$

where *W*_*s*_ is the wet weight of the swollen hydrogel in ethanol, *W*_*d*_ is the dried weight of the hydrogel, *ρ* is the density of ethanol, and *V* is the volume of the hydrogel.

The porous network structure was observed by scanning electron microscope (SEM, operating at 3 kV, TM3000, Hitachi) in the inner cross-section of lyophilized hydrogels. One hundred fifty microliters of each hydrogel in the buffer of phosphate buffered saline (PBS) was placed in a 2 mL centrifuge tube. The hydrogel was then incubated in 1.5 mL of PBS at 37 °C. The swelling ratio of the hydrogels was determined by checking the wet weight of each hydrogel after 24 h. The swelling ratio was calculated using the following equation: *Ws*/*Wi* × 100%, where *Wi* and *Ws* represented the wet weight of each initial hydrogel on day 0 and the wet weight of the swollen hydrogel after 24 h, respectively. The in vitro degradation of hydrogels was estimated by tracking the residual mass over time. The soaked hydrogels were retrieved from PBS buffer at defined time points, rinsed with DI water, and then freeze-dried. The remaining weight percentage was calculated by the Eq. (3):

3 $$remaining\ weight=\frac{W^{\prime }}{W_0}\times 100\%.\kern0.75em$$

where *W′* is the initial dried weight of the hydrogel and *W*_*0*_ is the dried weight of the hydrogel after incubation in pH 7.4 PBS at 37 °C at defined time points.

The rheological properties of the CMC/OTA (CO) and COA hydrogels were estimated at 25 °C using a rheometer (HR-2, Discovery Series Hybrid Rheometer, TA Instruments) with a cone and plate geometry of 40 mm diameter and a cone angle of 2°. The measurements of storage modulus (G’) and loss modulus (G”) were carried out at a frequency of 1 Hz and a dynamic strain of 1% against the gelling time. The dynamic strain sweep experiments of the hydrogels were investigated at a frequency of 1 Hz in the range of 0.1 to 800% dynamic strain amplitudes. The self-healing properties of equilibrium hydrogels were assessed by rheological experiments with continuous and alternating high strain (600%) and low strain (1%) as damage-healing cycles at a frequency of 1 Hz. The steady shear test was utilized to characterize the shear thinning behavior of the hydrogels, identified by measuring the viscosity in relation to the shear rate. The coherent small-angle X-ray scattering (SAXS) was used to investigate the changes of the nanoscale structure with the scattering vector (q)-range from 5 × 10^− 3^ Å^− 1^ to 3 × 10^− 1^ Å^− 1^ performed at about 8 keV of photon energy at the beamline station 25A1 of Taiwan Photon Source (TPS 25A1) at National Synchrotron Radiation Research Center (NSRRC) in Hsinchu, Taiwan.

### Neural stem cells (NSCs) cultured in hydrogels

Neural stem cells (NSCs) were derived from adult mouse brain as previously described [28]. The cell medium for NSCs was a mixture of high-glucose Dulbecco’s modified Eagle’s medium (HG-DMEM, Gibco, USA) and Ham’s F-12 (1:1) with extra ingredients of 10% fetal bovine serum (FBS, Gibco, USA), 400 μg/mL geneticin (G418, Invitrogen, USA), and 1% penicillin–streptomycin–amphotericin (PSA, Gibco, USA). NSCs were cultured in a humidified incubator at 37 °C and 5% CO_2_ with constantly refreshed culture media every 2 days. For three-dimensional cell culture in hydrogels, NSCs were first suspended in the 5 wt% CMC solution in twice concentrated culture medium with a cell density of 2 × 10^6^ cells/mL. Subsequently, the 400 ppm OTA@Au (or OTA) suspension in DI water was mixed with the cell suspended CMC solution in a 1:1 vol ratio for the preparation of the NSC-encapsulated hydrogel. The proliferation of NSCs in hydrogels was assayed by the Cell Counting Kit-8 (CCK-8) assay every 2 days for seven times, performed at a wavelength of 450 nm using the SpectraMax M5 plate reader.

The expression levels of neural-associated genes were evaluated using a KAPA SYBR Green qPCR kit (Kapa Biosystem, Inc., UK) through the real-time reverse transcriptase–polymerase chain reaction (RT-PCR) to assess the differentiation direction of NSCs embedded in hydrogels. The medium for NSCs in the cell differentiation evaluation was G418-free culture medium and the cell differentiation was assessed after 14-day culture. The data were obtained using a Step One Plus Real-Time PCR instrument (Applied Biosystems, USA). The markers of the nervous system employed in this research included nestin, glial fibrillary acidic protein (GFAP), β-tubulin, and microtubule-associated protein 2 (MAP2), and the related primer sequences are shown in Table S1 (Supporting information). Gene expression data were normalized against 3-phosphoglycerate dehydrogenase (GAPDH, the housekeeping gene) and then represented as relative proportions.

The protein expression levels of NSCs were further investigated by immunofluorescence staining of NSCs after 14 days. NSCs were fixed by 4 wt% paraformaldehyde (PFA, Sigma-Aldrich, USA) for 15 min at room temperature after removing medium and washed by 0.1 wt% Tween 20 in PBS (PBST) for three times. NSCs were blocked in a 2 wt% bovine serum albumin (BSA, Gibco, USA) solution (diluted with PBST) for 1 h with shaking at the speed of 60 rpm. Then, NSCs were incubated with mouse monoclonal anti-GFAP antibody (1:100, 14–9892-82, Invitrogen, USA) or rabbit monoclonal anti-MAP2 antibody (1:200, ab183830, Abcam, UK) at 4 °C overnight. On the next day, NSCs were washed by PBST and incubated with goat anti-mouse IgG H&L (Alexa Fluor®594, ab150116, Abcam, UK) or goat anti-rabbit IgG H&L (Alexa Fluor®594, ab150080, Abcam, UK) for 1 h at 25 °C. The cells nuclei were counterstained with the Hoechst 33258. The photomicrographs were captured with a fluorescence microscope (Nikon, Eclipse 80i, Japan) in association with a digital camera. The semi-quantitative data for average fluorescent intensity of GFAP- and MAP2-positive NSCs were calculated and acquired using ImageJ software.

### In vitro antioxidative and anti-inflammatory assay

The in vitro evaluation assay for antioxidative and anti-inflammatory properties were performed following the previous published literature [24]. NSCs were preliminarily treated with human interferon gamma (IFN-γ, 0.1 ng/mL, Peprotech, USA) for 12 h to trigger reactive oxygen species (ROS)-induced neuro-inflamed unhealthy status. The inflamed NSCs were collected using accumax (00–4666-56, Invitrogen, USA) and further cultured for 12 h with the CO or COA2 hydrogel to investigate the ability of the hydrogel to scavenge ROS and to rescue cells. Cells were stained with Solution 5 (ChemoMetec, Danmark) containing acridine orange (as the counterstain), propidium iodide (PI, defining dead cells), and VitaBright-48 (VB-48). Stained cells were analyzed for cell viability using the NucleoCounter® NC-3000™ system (ChemoMetec, Denmark). The rescue rate and dead rate were calculated by Eq. (4) and Eq. (5), respectively:

4 $$rescue\ rate=\frac{HR_h-{HR}_{ic}}{UH_{ic}-{UH}_{hc}}\times 100\%$$

5 $$dead\ rate=\frac{DR_h-{DR}_{ic}}{UH_{ic}-{UH}_{hc}}\times 100\%$$

where *HR*_*h*_ and *HR*_*ic*_ represent the healthy rate of hydrogel-treated group and single inflamed cells, respectively, *DR*_*h*_ and *DR*_*ic*_ represent the dead rate of hydrogel-treated group and single inflamed cells, respectively, and *UH*_*ic*_ and *UH*_*hc*_ represent the unhealthy rate of single inflamed cells and single healthy cells, respectively.

J774A.1 murine macrophages (ATCC, USA) were employed and cultured in HG-DMEM medium with 10% FBS and 1% PSA for investigating the anti-inflammatory ability. The cells were cultured in 24-well plates for 2 days in the incubator and the hydrogels were overlaid on cell seeding wells for 6 h to mimic the in vivo state of hydrogels in contact with macrophages. Collection of cells for further RT-PCR analysis was performed to ascertain the expression levels of inflammation-related genes, including interlukin-1β (IL-1β), interlukin-6 (IL-6), interlukin-10 (IL-10), and tumor necrosis factor (TNF-α), and the related primer sequences are shown in Table S2 (Supporting information). The level of expression was calculated and normalized to β-actin. Expression levels were computed and normalized to β-actin.

### In vivo rat model of Parkinson’s disease (PD) and the stereotaxic injection of hydrogels

Adult male Wistar rats (LASCO, Taiwan) weighing 250–350 g were employed in the present study for animal experiments. All procedures for animal experiments in this study were authorized by the Institutional Animal Care and Use Committee (IACUC) of the College of Medicine and Public Health, National Taiwan University (Approval number: 20210346) and complied with the guidelines for the care and use of laboratory animals. Rats received food and water freely when housed in a constant temperature and humidity environment with a 12-h light/dark cycle. The protocol for the induction of Parkinson’s disease (PD) in rats by microinjection of 6-hydroxydopamine (6-OHDA, as neurotoxin, Sigma-Aldrich, USA) into the unilateral substantia nigra pars compacta (SNpc) was previously reported in the literature [29].

Rats were anesthetized with the veterinary anesthetic (Zoletil™ 50, 20 to 40 mg/kg, Virbac, France) and mounted in a stereotactic frame (Kopf Instruments). 6-OHDA (4 μL, each, 2 wt% in saline) were injected using a stainless-steel cannula connected to a 30G Hamilton microsyringe through polyethylene tubing controlled by an infusion pump. Coordination of Paxinos- and Watson-based rat brain atlas [30], the injection site was selected at anterior-posterior (AP) -2.8 mm × lateral (LAT) -2.0 mm × depth (DEP) -8.0 mm at a rate of 0.5 μL/min. An additional 5 min is required before the injection cannula is withdrawn to reduce the loss of fluid dragged out along the injection tract. All rats were evaluated through the rotational behavior test by extra injection of apomorphine (Sigma-Aldrich, USA) to judge the effect of lesion in the model [31]. An indicator of successful PD induction was that PD rats had more than 25 turns every 5 min after SNpc injection of 6-OHDA, consistently turning to the contralateral side of the lesion.

Those PD rats successfully induced after confirmation by behavioral tests were selected to undergo single-sided injections of saline and hydrogels, i.e., CO hydrogel, COA2 hydrogel, and COA2 hydrogel loaded with curcumin (10 μM, Sigma-Aldrich, USA). Rats were anesthetized with Zoletil™ 50 and head-fixed to a stereotaxic apparatus (Kopf Instruments). The injection location was consistent with the lesion site. The sterile saline, the CO hydrogel, the COA2 hydrogel, or the curcumin-loaded COA2 hydrogel with 4 μL was injected via a Hamilton microsyringe with a 26-gauge needle at the rate of 0.5 μL/min. The needle was slowly withdrawn after a delay of 5 min to prevent reflux.

### Behavior tests and electrophysiological analyses

The behaviors of rats were tested at both 7 and 14 days by subcutaneous injection of apomorphine (0.05 mg/kg, 10 mg dissolved in 0.2 wt% ascorbic acid aqueous solution). The spontaneous rotational behavior of the rats was documented for 5 min and the mean speed of each rotation was further calculated to assess the reduction of PD symptoms. The forelimb lateralization was also investigated using the cylinder asymmetry test, taking advantage of the naturally explorative nature of rodents for new environments. Rats were separately positioned in a glass cylinder (22 cm in diameter and 26 cm in height). After the rats first contacted the cylinder wall with the impaired or unimpaired forelimbs, the rats were videotaped for 5 min and the number of contacts between the two forelimbs was counted to facilitate comparison with healthy rats.

The electrophysiological analysis was conducted by following literature with some changes [32]. Rats were anesthestized with Zoletil™ 50 and placed in a stereotactic frame (Kopf Instruments). The skull and dura mater covering the subthalamic nucleus (STN) were then carefully removed, and several 30-s extracellular recordings were performed in the STN. The 8-channel microelectrode array (Kedou BC Inc., China) was vertically inserted into the position of the STN counterpart at AP-3.8 mm × LAT − 2.4 mm × DEP − 6.5 mm based on Paxinos and Watson’s rat brain atlas. The extracellular neuronal activity was magnified by a preamplifier (Model 3600, A-M system LLC., USA), displayed on an oscilloscope, and stored in a computer equipped with a data acquisition interface (Power1401-3A, CED LTD., UK). The signal was recorded with no interference. A signal-to-noise ratio greater than 3:1 was recorded only for neuronal activity and available for further investigation. The overall spike rate was calculated using the Spike 2 software (CED LTD., UK).

### Immunofluorescent and immunohistochemical staining

PD rats were sacrificed followed by perfusing the heart with saline and then fixing with 4 wt% PFA for further staining analysis. The brains were taken out and soaked in 4 wt% PFA overnight for secondary fixation. The brains were dehydrated, wrapped in paraffin, and sectioned at 4 μm. Following deparaffinization, the slices were washed with PBST followed by blocking with 2 wt% BSA solution for 30 min. The brain sections were incubated at 4 °C overnight with various primary antibodies for different purpose including in vivo biocompatibility and histological evaluation for PD. The primary antibodies used in this study involved Anti Iba1 (Rabbit, for immunocytochemistry, 019–19,741, 1:500, Wako Pure Chemical Industries, Ltd., Japan), ABclonal rabbit polyclonal anti-CD86 antibody (A1199, 1:200, ABclonal Inc., USA), ABclonal rabbit polyclonal anti-CD163 antibody (A8383, 1:200, ABclonal Inc., USA), recombinant anti- tyrosine hydroxylase (TH) antibody (EP1533Y/ab75875, 1:50, Abcam, UK), and mouse monoclonal anti-GFAP antibody (14–9892-82, 1:100, Invitrogen, USA). For anti-Iba1 staining, the tissue sections were then developed using the diaminobenzidine reaction. For other antibodies, the secondary antibodies were applied and incubated with the brain sections at 25 °C for 1 h, including goat anti-rabbit IgG H&L (ab150077, 1:500, Alexa Fluor® 488), goat anti-rabbit IgG H&L (ab150080, 1:500, Alexa Fluor® 594), and secondary alpaca anti-mouse IgG1 VHH Alexa Fluor 488 antibody (sms1AF488–1, 1:500, Proteintech). The photomicrographs were captured with a fluorescence microscope (Nikon, Eclipse 80i, Japan) in association with a digital camera and quantification data for average fluorescent intensity of these photomicrographs were calculated using the ImageJ software.

### Statistical analysis

All quantitative results were acquired by independent experiments and at least three replications were performed to exclude unexpected circumstances. Computed data are exhibited as mean ± standard deviation. Statistical differences between groups were carried out utilizing the commercially distributed packaged GraphPad Prism 9 as well as the Student’s t-test, and values were deemed statistically meaningful if *p* < 0.05.

## Results

### Synthesis and characterization of OTA and OTA@Au

**Fig. 1:**
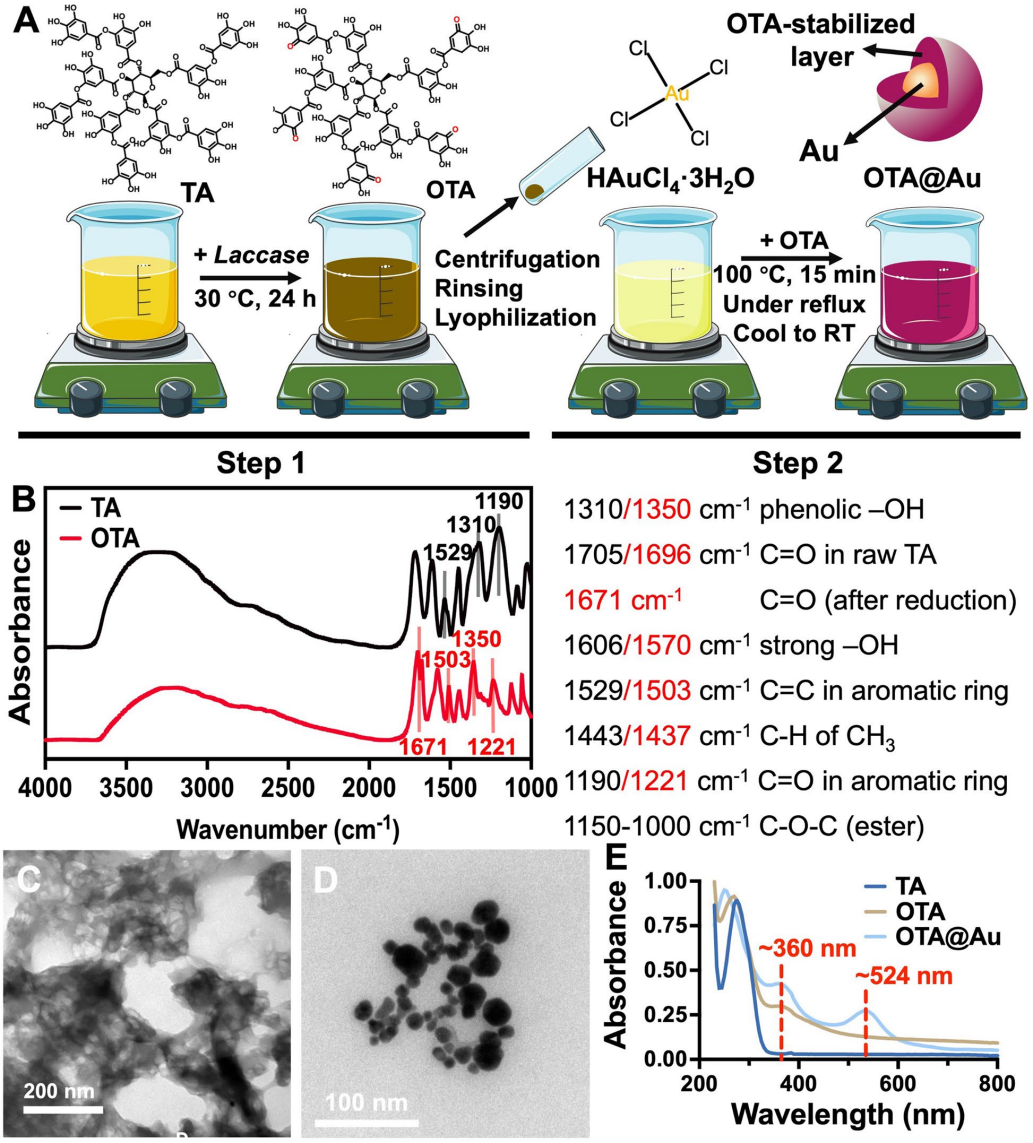
Preparation and characterization of oxidized tannic acid (OTA) and oxidized tannic acid modified gold nanoparticles (OTA@Au). **A** The synthetic diagram of OTA@Au nano-crosslinkers. **B** The IR spectra of tannic acid (TA) and OTA. **C** The TEM image of OTA. **D** The TEM image of OTA@Au. **E** The UV-visible spectra of TA, OTA, and OTA@Au

The quinone-functionalized OTA and the conductive OTA@Au nanoparticle were synthesized following the synthetic routes demonstrated in Fig. 1A. The IR spectra of TA and OTA are demonstrated in Fig. 1B, which verified the existence of carbonyl groups in OTA. For the pristine TA, the absorption bands in the wavenumber range 3000–3700 cm^− 1^ and near 1310 cm^− 1^ were ascribed to the vibration of the phenolic hydroxyl groups. The stretching vibration of C═O and C═C on the aromatic rings of TA showed up at 1190 and 1529 cm^− 1^, respectively. Absorption peaks in the range of 1000–1150 cm^− 1^ mainly referred to the ether bond in the structure of TA. In OTA, the laccase oxidation converted the phenols into active *o*-quinones [33]. The absorbance peak of OTA at 1671 cm^− 1^ was assigned to the stretching of conjugated carbonyl groups, and such stretching was associated with the formation of a quinone in the intermediate step. Meanwhile, the symmetric structure of the hydroxyl group on the pristine TA underwent transformation due to the enzymatic oxidation by laccase, with characteristic peaks slightly shifted by the corresponding change in the intramolecular hydrogen bonding [34]. The OTA used in this work showed the color of yellowish brown and owned a carbonyl content of 0.348 ± 0.009%. Images of Fig. 1C from TEM revealed that OTA was in the form of random polymer clusters without a specific shape. During the synthetic process of OTA@Au, the color of the solution changed from pale yellow to uniformly deep purplish-red under boiling conditions after the addition of OTA. The spherical-like shape of OTA@Au nanoparticles was clearly seen by TEM with an average diameter of 27.79 ± 2.89 nm in Fig. 1D. The polymeric chain of CMC was also observed by TEM, as displayed in Fig. S1 (Supporting information). The value of the carbonyl content of the OTA@Au nano-crosslinker was 0.395 ± 0.025%, slightly higher than that of pristine OTA. Besides, the UV-Vis spectrum (Fig. 1E) of OTA showed an absorbance peak at 360 nm, while such a peak was not detected in the UV–Vis spectrum of the pristine TA. The UV-Vis peak at 360 nm may be associated with the quinone groups on OTA. Moreover, OTA@Au exhibited absorbance peaks at both ~ 360 nm and ~ 524 nm, indicating the presence of the AuNPs and carbonyl groups.

### Preparation and optimization of self-healing hydrogels

The bioactive self-healing hydrogel was prepared from mixing CMC (main chain) and OTA@Au (nano-crosslinker) through dynamic Schiff reaction at 25 °C. The average hydrodynamic diameter and ζ-potential of CMC and OTA@Au are shown in Table S3 (Supporting information). The ζ-potential values of the OTA@Au dispersion and the CMC aqueous solution and were − 45.6 ± 2.7 mV and − 38.3 ± 5.4 mV, respectively, suggesting the stability of the components. The average hydrodynamic diameter of OTA@Au was 30.26 ± 1.79 nm, which was ~ 10% larger than the particle size observed from TEM and probably caused by the external hydration layer of OTA@Au. Various hydrogels were prepared with different formulae of CMC and OTA@Au, and the component ratios together with abbreviated names of hydrogels are listed in Table S4 (Supporting information). Considering the gelation time, three COA hydrogels and the CO hydrogel (control group) were selected for detailed basic property evaluation, including the measurement of self-healing time, porosity, conductivity, gel fraction, swelling ratio, and storage modulus. The evaluation results are summarized in Table 1. Additionally, the macroscopic images of the hydrogels are shown in Fig. S2 (Supporting information). With the increasing concentration of OTA@Au, the color of the transparent hydrogels transformed from light pink (COA3 hydrogel) to deep purplish-red (COA1 hydrogel), in contrast to the CO hydrogel with transparent brown color. The conductive values of all COA hydrogels were significantly higher than the non-Au-containing CO hydrogel and the value of conductivity enhanced as the concentration of OTA@Au nano-crosslinkers increased, i.e., 1.15 ± 0.10, 1.36 ± 0.14, and 1.43 ± 0.18 mS/cm for COA hydrogels with 150, 200, and 250 ppm OTA@Au. The conductivity performance of COA hydrogels was also examined before damage and after self-healing to validate the stability and consistency of the conductivity values. Overall, a higher concentration of OTA@Au nano-crosslinker in the hydrogel resulted in shorter gelation time, faster self-healing time, lower porosity, greater gel fraction, and higher storage modulus. However, OTA@Au with the high concentration of 250 ppm would crosslink CMC too rapidly that mixing was not possible to obtain a completely homogeneous COA1 hydrogel for subsequent applications. On balance, the uniform COA hydrogel selected for further studies contained 2.5 wt% CMC and 200 ppm OTA@Au (i.e., the hydrogel COA2 in Table S4 and Table 1).

**Table 1:** Basic properties of the hydrogels with different formulae

Abbreviated name	Self-healing time (min)	Porosity (%)	Conductivity (mS·cm^**− 1**^)	Gel fraction (%)	Swelling ratio (%)	Storage modulus (Pa)
CO	~ 22	84.6 ± 5.4	0.30 ± 0.01	84.21 ± 4.44	157.1 ± 25.6	~ 150
COA1	~ 8	90.5 ± 2.1	1.43 ± 0.18	95.83 ± 2.33	112.4 ± 16.7	~ 210
COA2	~ 12	92.4 ± 1.3	1.36 ± 0.14	94.72 ± 1.91	117.0 ± 11.1	~ 180
COA3	~ 30	94.1 ± 2.9	1.15 ± 0.10	90.62 ± 2.76	123.6 ± 20.2	~ 120

### Characteristics of self-healing hydrogels

**Fig. 2:**
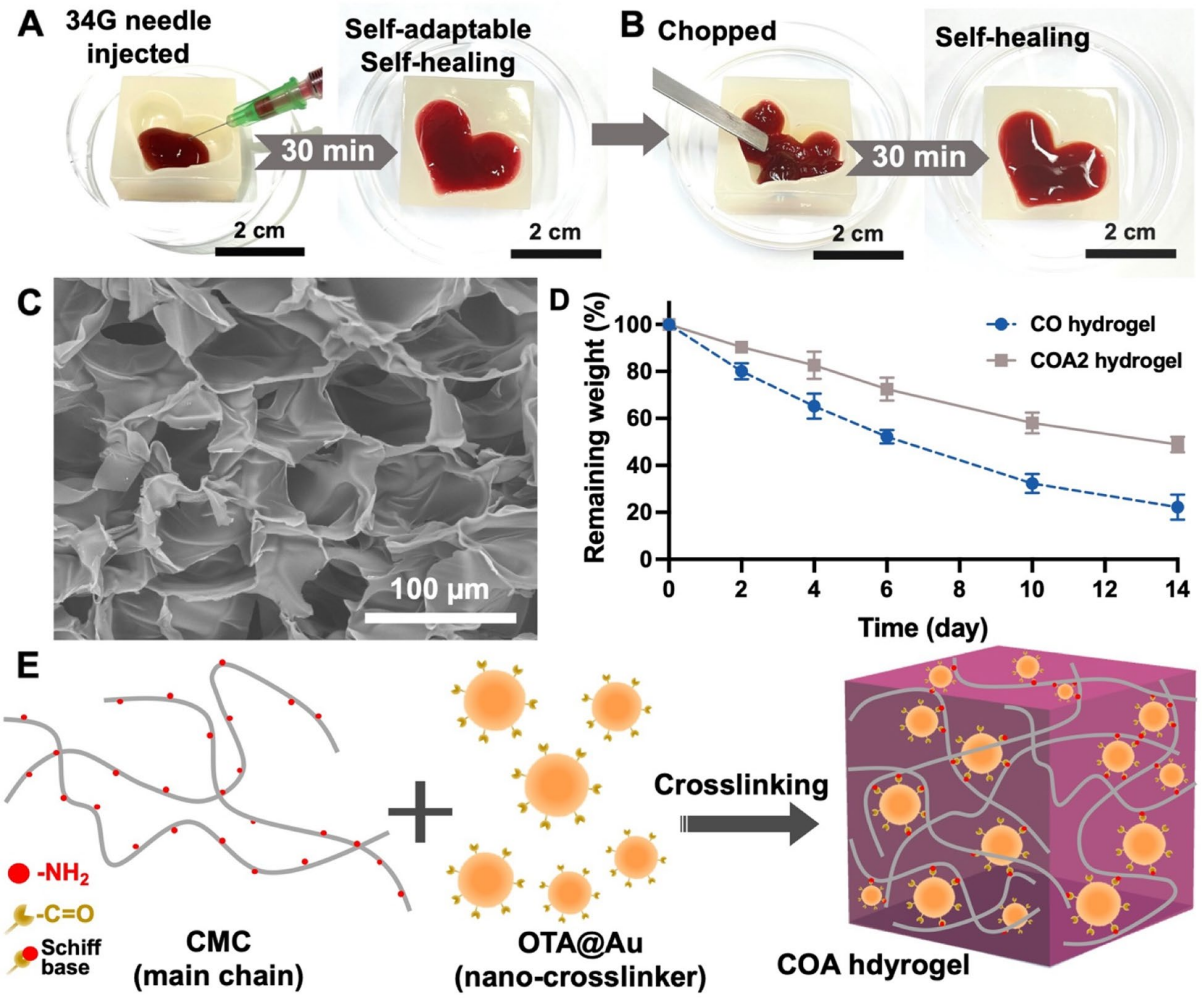
Preparation and morphology of the self-healing hydrogels. **A** The COA hydrogels can be injected through 34-gauge syringe needles with 80 μm internal diameter into the heart-shaped mold (**A**). An integrated heart-shaped hydrogel with smooth and homogenous appearance formed after self-adaption and self-healing for 30 min at 25 °C. **B** The integrated hydrogel was chopped into small pieces and then refilled the heart-shaped mold. The heart-shaped hydrogel self-healed for 30 min and recovered into its original shape. **C** The SEM image for the cross-section of the COA2 hydrogel. **D** Remaining weights of the CO and COA2 hydrogel in PBS at 37 °C. **E** Schematic diagram for the possible gelation mechanism of COA hydrogels

COA hydrogels demonstrated injectability and self-healing ability. COA2 hydrogel was extrudable through a 34-gauge needle (80 μm internal diameter) into a heart-shaped mold and subsequently formed an integrated hydrogel with smooth and homogenous appearance after 30 min due to self-adaptive and self-healing capabilities, as shown in Fig. 2A. In addition, the bulk COA2 hydrogel were further chopped into pieces and repopulated with the mold of heart shape. The hydrogel self-healed and reverted into the former heart shape within 30 min (Fig. 2B). The outstanding self-healing and self-adaptive properties of the hydrogel were proven in these macroscopic experiments. The SEM images of the COA2 and CO hydrogel are shown in Fig. 2C and Fig. S3 (Supporting information), respectively, and the quantitative data, i.e., pore size and wall thickness, are summarized in Table S5 (Supporting information). The average internal pore size of CO hydrogel (76.12 ± 9.30 μm) was larger than that of COA2 hydrogel (59.72 ± 6.70 μm), and the wall thickness of CO hydrogel (2.01 ± 0.25 μm) was thicker than that of COA2 hydrogel (1.59 ± 0.27 μm). There was significant difference in the porous structure of the two hydrogels. Besides, CO hydrogel showed a faster degradation rate than COA2 hydrogel within 14 days (Fig. 2D), probably due to the lower chemical crosslinking density (gel fraction in Table 1). According to the structure difference, a possible gelling mechanism of the COA hydrogel is demonstrated in Fig. 2E.

**Fig. 3:**
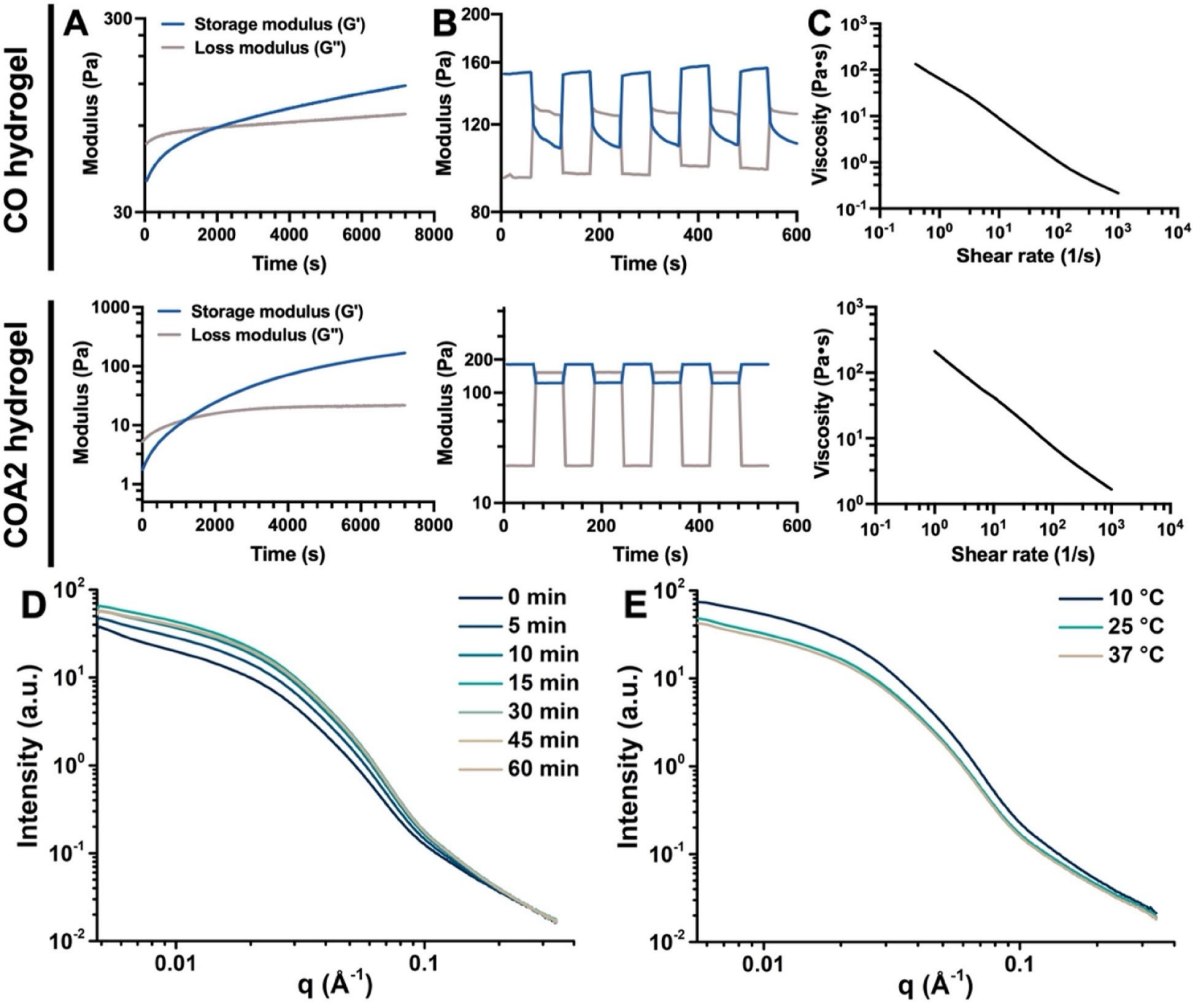
Rheological analyses and time-resolved SAXS experiments of the self-healing hydrogels. **A** Time-sweep experiments showing the storage moduli (G’) and loss moduli (G”) of the hydrogels against the gelling time at 1 Hz frequency and 1% dynamic strain. **B** Self-healing experiments showing the G’ and G” values of the equilibrium hydrogels at 1 Hz frequency and alternate continuous damage-healing cycles of 1 and 600% dynamic strains at 25 °C. **C** The steady shear-thinning properties of the hydrogels determined by measuring the viscosity versus shear rate. **D** Time-dependent coherent SAXS profiles acquired during the gelation process at 25 °C for investigation of microstructure changes of the hydrogel upon crosslinking. **E** SAXS profiles for the hydrogel at different temperatures. The SAXS data were obtained from COA2 hydrogel

The mechanical properties of the soft hydrogels were examined through rheological measurements at 25 °C by a rheometer. The G’ and G” of the hydrogels against the gelling time are demonstrated in Fig. 3A. The COA2 hydrogel exhibited significantly shorter gelling time than CO hydrogel. The stabilized G’ of CO hydrogel and COA2 hydrogel was ~ 150 Pa and ~ 180 Pa, respectively. Fig. S4 (Supporting Information) shows the shear strain-induced modulus variations for CO and COA2 hydrogels over a dynamic strain amplitude range of 0.1 to 800% at 1 Hz frequency, showing that the gel-to-sol conversion happened at ~ 540% and ~ 560% strain for CO and COA2 hydrogels, respectively. These hydrogels demonstrated reversible gel-sol-gel transitions in successive strain-induced damage-healing cycles at alternating strains of 1 and 600% (Fig. 3B). When the larger strain (600%) was applied, the hydrogels was converted from a gel-like state (G’ over G”) into a sol-gel-like state (G” over G’). Following the administration of a lower strain (1%), the G’ of the hydrogel returned to the initially gel state. The COA2 self-healing hydrogel recovered the structure almost completely even after several repeated cycles, in comparison to the CO hydrogel recovered with fluctuating values. The steady shear viscosity of each hydrogel illustrated in Fig. 3C suggested that both CO and COA2 hydrogels had shear-thinning property and favorable injectability.

The nanostructure of COA2 hydrogel was characterized using coherent SAXS. The SAXS profile showed a spheroid form factor in the COA2 hydrogel (shown in Fig. 3D). The SAXS profiles of each component alone are displayed in Fig. S5 (Supporting information). The intensity of COA2 hydrogels increased with the prolonged gelation time, proposing that a more extensive fractal network was generated in the low-q region (q ∼ 5 × 10^− 3^ Å^− 1^) [35]. The SAXS profile of COA2 hydrogel did not change further after 15 min, implying that such hydrogel had relatively stable crosslinking after 15 min, which was consistent with the finding of rheological tests. Also, OTA@Au had an obvious spherical structure with the formal factor of the nanoparticulate COA hydrogel system was mainly contributed by OTA@Au [36]. The form factor of the COA2 hydrogel had no significant change with increasing crosslinking time, which indicated a possible good retention of the spherical structure of OTA@Au via Schiff base linkage during the crosslinking process. Meanwhile, the SAXS profiles for the equilibrium COA2 hydrogel at different temperatures were recorded and are shown in Fig. 3E. The changes in intensities were attributed to the extent of hydrogen bonding of OTA@Au which was strongly influenced by the temperature and thus affected the network structure of the hydrogel. The results of nanostructure evolution further corroborate the previously proposed hypothesis of crosslinking mechanism for the COA hydrogel system.

### NSCs cultured in hydrogels

**Fig. 4:**
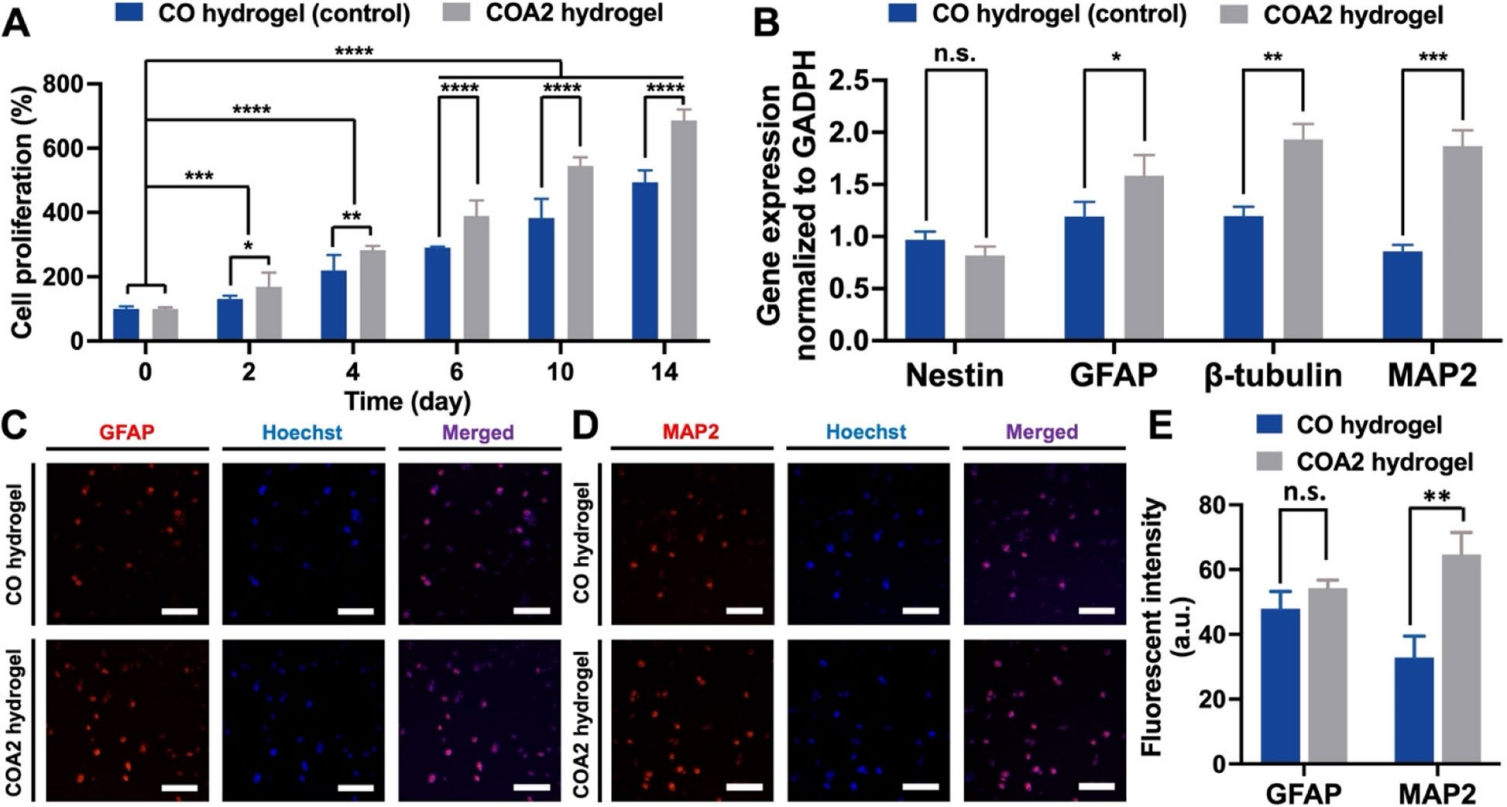
Proliferation, differentiation, and immunofluorescent staining of neural stem cells (NSCs) encapsulated in the hydrogels. **A** Cell proliferation of NSCs embedded in the CO and COA2 hydrogels measured by CCK-8 assay during a culture period of 14 days. The proliferation was normalized to the value at day 0 and expressed as the percentage of the cell proliferation (%). **B** Expression of neural-related genes in NSCs encapsulated in the hydrogels were analyzed by RT-PCR at 14 days. The expression levels normalized to that of housekeeping gene (GAPDH) and represented by the relative ratios of gene expression. Immunofluorescent images of NSCs stained for (**C**) GFAP and (**D**) MAP2 at 14 days. Scale bars represent for 100 μm. **E** Quantification of the fluorescent intensity in GFAP- and MAP2-positive NSCs. *p < 0.05, **p < 0.01, and ***p < 0.001 between the indicated groups

The proliferation of NSCs encapsulated in the CO or COA2 hydrogel was analyzed by CCK-8 assay for a period of 14 days to assess the cytocompatibility, as shown in Fig. 4A. The COA2 hydrogel showed the greater cell proliferation rate (in the value of ∼686%) than the CO hydrogel (in the value of ∼494%) at 14 days, and significant differences were observed between these two groups during each time point of the evaluation within 14 days. A previously reported CMC-based conductive hydrogel crosslinked with dialdehyde polyurethane containing nanogold was additionally selected as a positive control group to evaluate the ability of COA2 hydrogel to promote the proliferation of NSCs. As shown in Table S6, NSCs in the COA2 hydrogel exhibited a slightly higher proliferation rate than that of the positive control group (~ 629%), confirming the promotive effect of the COA2 hydrogel on cell proliferation.

The gene and protein expression of NSCs embedded in hydrogels were characterized with RT-PCR and immunofluorescence staining (Fig. 4B-D). The gene expression level of nestin did not differ significantly in CO and COA2 hydrogels. The NSCs grown and differentiated in the hydrogel for 14 days maintained the round shape without the stretched morphology of dendrites or axons. NSCs laden in the hydrogel were subjected to the force from all directions in the 3D environment and may not easily extend their morphology. Some tiny protrusions on spherical NSCs were noticed after 14 days of culture, but the small morphological change was invisible after staining. The expression levels of GFAP (glial marker), β-tubulin (early neuronal marker), and MAP2 (mature neuronal marker) were all upregulated significantly in COA2 hydrogels compared with the CO hydrogel after 14 days. The immunofluorescence staining of NSCs was presented 14 days later in CO hydrogels and COA2 hydrogels for GFAP (Fig. 4C) and MAP2 (Fig. 4D) to verify the results of gene expression. MAP2-positive and GFAP-positive NSCs were noticeably found in both hydrogels. The semi-quantitative data for average fluorescent intensity of GFAP- and MAP2-positive NSCs were further calculated to confirm the differentiation states and are summarized in Fig. 4E. The protein expression of MAP2 was apparently increased in the COA2 hydrogel, suggesting that NSCs cultured in the COA2 hydrogel underwent potential differentiation towards neuronal cells.

### Antioxidative and anti-inflammatory capabilities of hydrogels

**Fig. 5:**
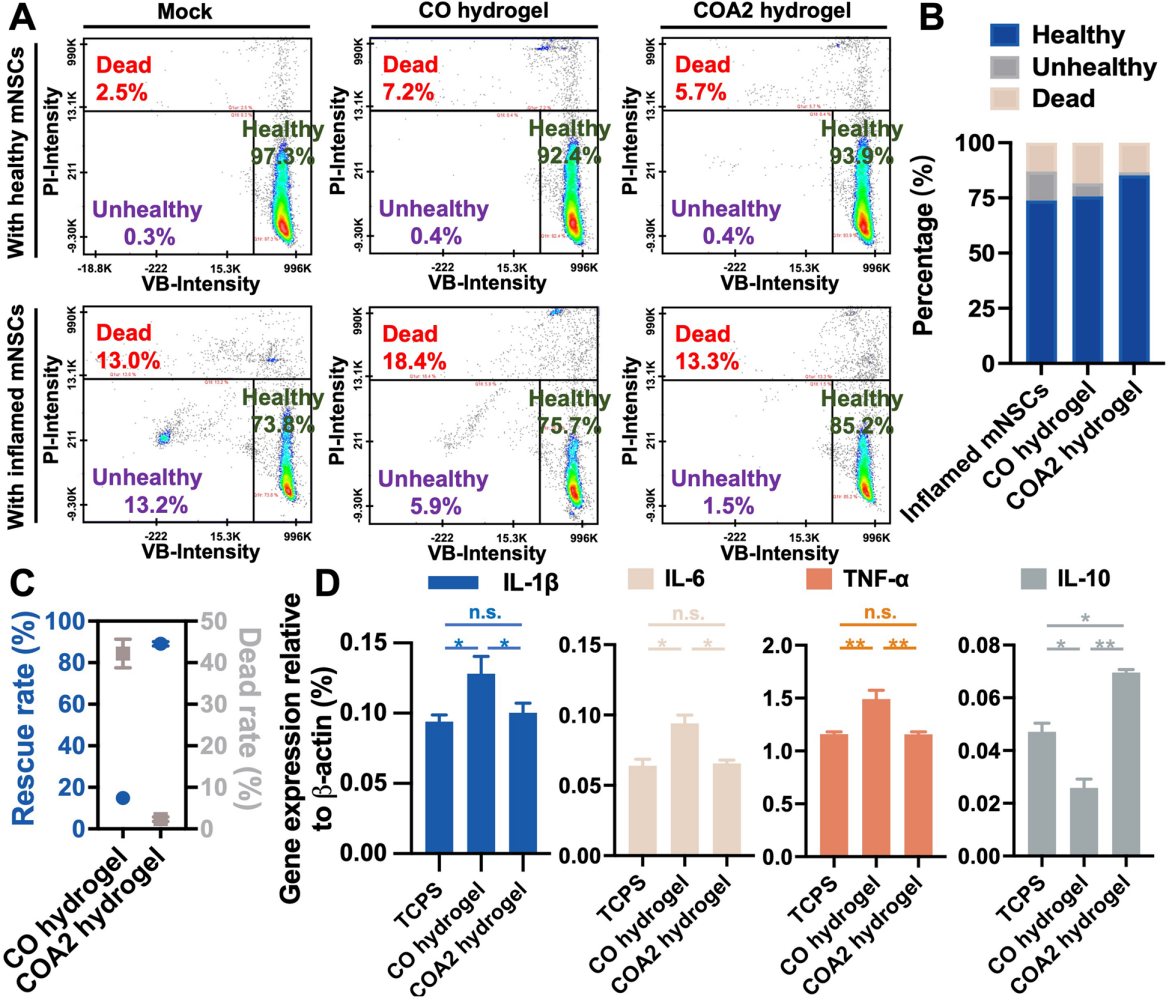
In vitro antioxidation and anti-inflammatory evaluations of the hydrogels. **A** Cell viability tests for either healthy or inflamed NSCs. Scatter plots showed changes in propidium iodide (PI) and Vitabright-48 (VB-48) staining in response to the cell treatment with encapsulation by the CO hydrogel and COA2 hydrogel compared to the single cells (mock). **B** Summary for the distribution of inflamed NSC status after the treatment of CO hydrogel or COA2 hydrogel compared to the single inflamed cells (mock). **C** Quantification for the rescue rate and dead rate of the inflamed NSCs after the treatment of the CO hydrogel or COA2 hydrogel for 12 h. **D** Gene expression of the J774A.1 macrophages analyzed by RT-PCR after contacting the CO hydrogel or COA2 hydrogel for 6 h compared to macrophages cultured on the tissue-culture polystyrene plate (TCPS) in vitro. The expression levels were normalized to that of β-actin and represented by the relative ratios of gene expression. *p < 0.05 and **p < 0.01 between the indicated groups

The antioxidant capacity of COA2 hydrogel was assessed using a previously published in vitro inflammatory NSC assay by quantifying the capability to scavenge ROS and rescue inflamed NSCs [24]. The VB-48 assay was applied to determine the cellular status pre- and post-treatment with the human IFN-γ as well as to study a further unhealthy/death ratio of inflamed NSCs embedded in two different hydrogels for 12 h, as shown in Fig. 5A. The distribution of cell health states was quantified as a graph illustrated in Fig. 5B, while the rescue rate and the dead rate were defined and calculated as summarized in Fig. 5C. The percentages of unhealthy and dead NSCs were significantly increased in IFN-γ-treated inflamed NSCs (~ 13.2% for unhealthy and ~ 13% for dead) compared to those in control healthy NSCs (~ 0.3% for unhealthy and ~ 2.5% for dead), indicating ROS production and neuroinflammation [37]. The percentages of unhealthy inflamed NSCs after 12 h of encapsulation in either hydrogel decreased notably, especially for COA2 hydrogel (i.e., ~ 5.9% for CO hydrogel and ~ 1.5% for COA2 hydrogel). There were significant differences between CO and COA hydrogels in both the rescue rate and dead rate for inflamed NSCs. Nearly 90% of unhealthy inflamed NSCs after 12 h of encapsulation by COA2 hydrogel were recovered to the healthy status, which was much greater than only ~ 15% rescue rate by CO hydrogel. These differences supported the effective rescue function for inflamed NSCs in vitro by COA2 hydrogel. Meanwhile, the dead ratio of inflamed NSCs was ~ 42% in CO hydrogel and only ~ 2% in COA2 hydrogel, confirming the superior anti-inflammatory property of COA2 hydrogel.

In vitro macrophage responses were also employed to assess the anti-inflammatory properties of hydrogel [24], through examination of the inflammation-related gene expression in J774A.1 macrophages by RT-PCR. Findings are revealed in Fig. 5D. Macrophages exposed to CO hydrogel for 12 h had significantly increased expression of IL-1β, IL-6 and TNF-α genes compared to both COA2 hydrogel and tissue culture polystyrene (TCPS) control. COA2 and TCPS groups did not express proinflammatory genes differently. Besides, COA2 and TCPS groups showed higher gene expression levels of IL-10, an important anti-inflammatory factor, than the CO group. Meanwhile, the IL-10 gene expression was significantly upregulated by the COA2 hydrogel vs. the control TCPS. These data further supported the anti-inflammatory property of COA2 hydrogel.

### Efficacy evaluation by the PD rat model

**Fig. 6:**
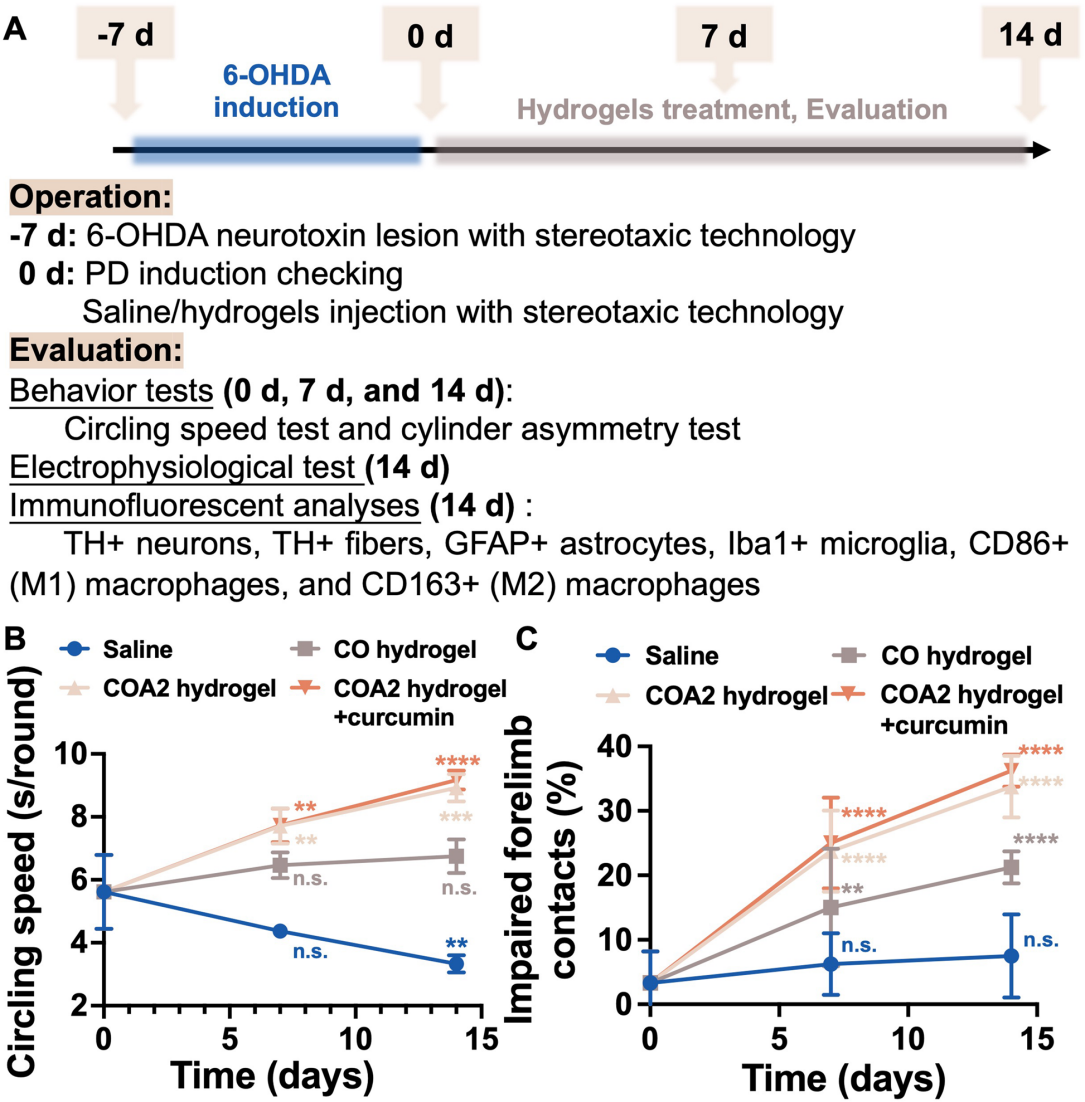
Behavior tests of Parkinson’s disease (PD) rats treated by hydrogels. **A** Schematic illustration of the PD rat model. 6-hydroxydopamine (6-OHDA) as a neurotoxin was utilized to induce PD through injection into the brains of rats. After confirmation of PD induction, the hydrogels were further injected into the lesion regions for 14 days, and the efficacy of treatment was evaluated through behavior tests, electrophysiological tests, and immunofluorescent analyses. **B**, **C** Quantitative assessment for the functional recovery of different condition treated PD rats (injected by saline, CO hydrogel, COA2 hydrogel, or COA2 hydrogel loaded with curcumin) as compared to the untreated PD rats (0 day), based on the left-side circling speeds (**B**) and the impaired forelimb contact proportion (**C**). **p < 0.01, ***p < 0.001, and ****p < 0.0001 between the indicated groups

A schematic representation of the potential ability of COA hydrogel to treat PD using the 6-OHDA-induced PD rat model is demonstrated in Fig. 6A, where the typical image of injected 6-OHDA neurotoxin lesion is shown in Fig. S6A (Supporting information). Two behavioral tests, including the circling speed test (Fig. S6B, Supporting information) and the cylindrical asymmetry test (Fig. S6C, Supporting information), were used to assess motor function recovery in treated PD rats. The results are presented in Fig. 6B and C. After 14 days of injection, PD rats treated with COA2 hydrogel or with curcumin-loaded COA2 hydrogel showed a significant increase in counterclockwise rotation time per round, accompanied by some heterolateral groping. For CO hydrogel-treated PD rats, there was no significant difference in circling behavior between day 0 and day 14. By contrast, the saline-treated PD rats showed continuous left-side rotational behavior that deteriorated with time. All three hydrogel-treated groups showed substantial improvement in the contact ratio of the impaired forelimbs compared to the saline-treated group. Both COA2 hydrogel and curcumin-loaded COA2 treated groups regained greater than 30% of the forelimb contact, and no statistical difference between the two groups was observed. Meanwhile, the control saline-treated group did not show any significant recovery of motor function based on the ratio of the impaired forelimb usage.

**Fig. 7:**
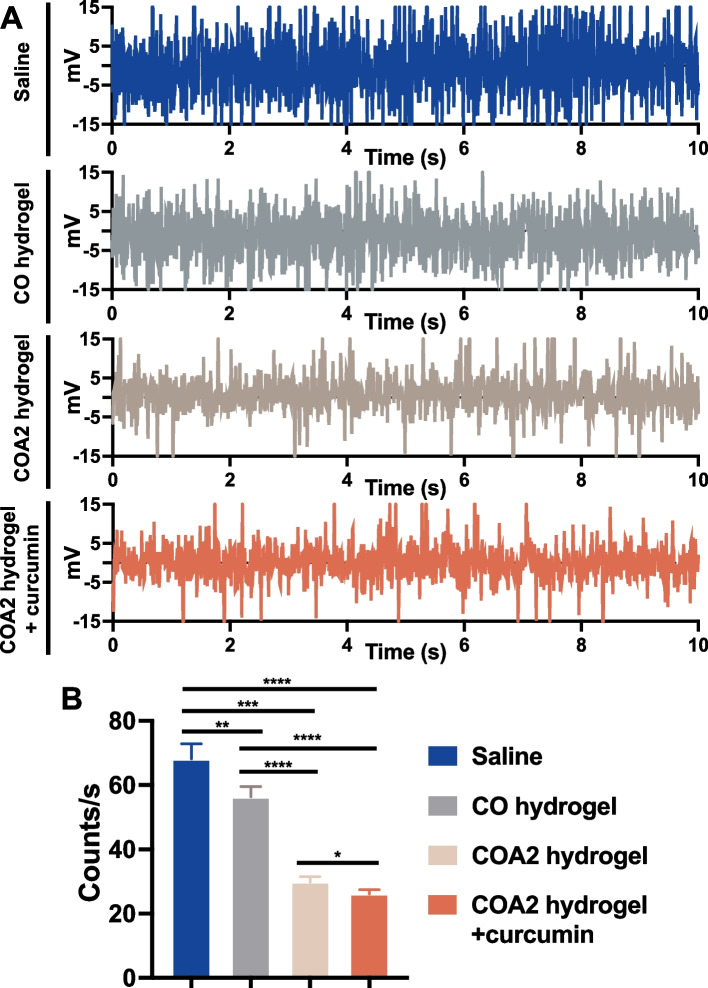
Electrophysiological analyses of PD rats treated by hydrogels. **A** Electrophysiological traces of subthalamic nucleus (STN) spikes in different condition treated PD rats within 10 s at 14 days. **B** Quantitative overall spike rates of each group from a 30-s period of each recording devoid of significant noise at 14 days. *p < 0.05, **p < 0.01, ***p < 0.001, and ****p < 0.0001 between the indicated groups

Electrophysiological measurement was performed to investigate the abnormal discharge behavior of projection neurons in the STN region of the brain after different treatments (Fig. S6D, Supporting information), and the results are displayed in Fig. 7A. The single-unit recordings in the STN revealed that in all four groups there existed burst firing which was one of the specific landmarks for PD. The irregular discharge phenomenon was obviously alleviated in hydrogel-treated groups vs. the saline-treated group, particularly in COA2 and curcumin-loaded COA2 groups. The overall spike rates were quantified and the results are summarized in Fig. 7B. The COA2 hydrogel and curcumin-loaded COA2 hydrogel groups showed significantly lower spike counts (29.80 ± 1.71 spikes/s and 26.18 ± 1.28 spikes/s, respectively) than the other two groups. The COA2 hydrogel group had the lowest spike counts. The electrophysiological measurement supported that injection of either COA2 hydrogel or curcumin-loaded COA2 hydrogel enhanced the functional recovery of PD rats.

**Fig. 8:**
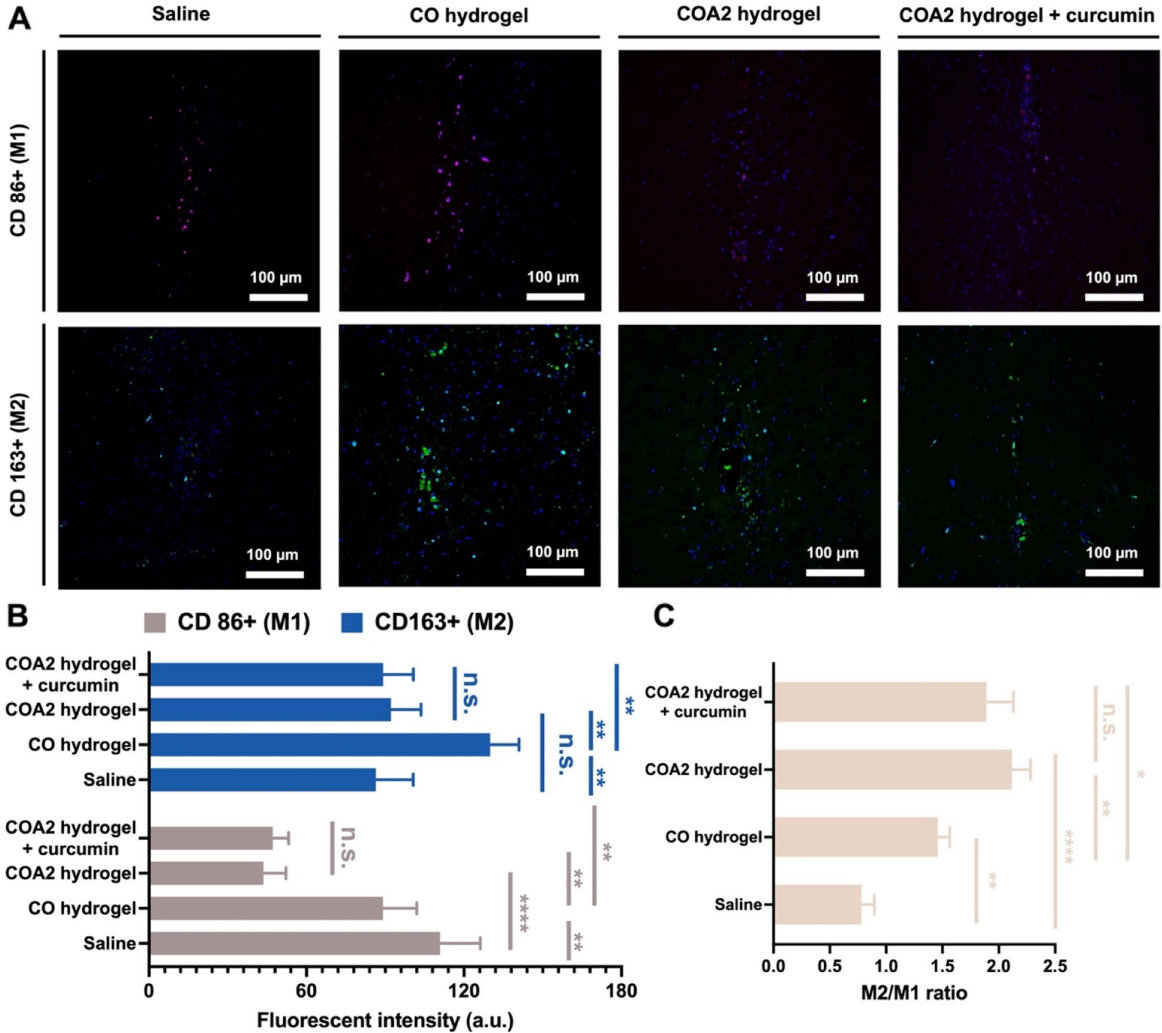
Biocompatibility evaluation of the hydrogels for treating PD rats. **A** In vivo immunofluorescent images of CD86 positive (CD86+) M1 macrophages (red) and CD163 positive (CD163+) M2 macrophages (green) for the explanted tissue after implantation in the brain for 14 days. Quantification for (**B**) the fluorescent intensity of M1 and M2 macrophages and (**C**) the ratios of M2/M1 in vivo. *p < 0.05, **p < 0.01, and ****p < 0.0001 between the indicated groups

The in vivo anti-inflammation and biocompatibility of the hydrogel were confirmed by immunohistochemical and immunofluorescent staining of microglia and M1/M2 type macrophage distribution in histological sections of brain tissue, as shown in Fig. S8 (Supporting information) and Fig. 8A, respectively. The hydrogel (4 μL) injected to the target site of the rat brains was almost degraded after 14 days for each hydrogel. The amount of microglia in the brains of PD rats implanted with COA2 hydrogel and curcumin-loaded COA2 hydrogel was dramatically reduced compared to the other groups. Besides, the group implanted with CO hydrogel showed slightly fewer microglia than the saline-treated control group. Quantification of the mean fluorescence intensities of M1 and M2 macrophages (Fig. 8B) revealed that COA2 hydrogel and curcumin-loaded COA2 hydrogel groups had significantly less M1 and M2 macrophages than the COA hydrogel group. Meanwhile, the M2/M1 ratio (Fig. 8C) was also significantly higher in COA2 hydrogel and curcumin-loaded COA2 hydrogel groups vs. the CO hydrogel group. There were no significant differences observed between the COA2 and curcumin-loaded COA2 hydrogel groups, either in intensities or M2/M1 ratios. The M2/M1 ratios of all hydrogel groups (~ 1.46 for CO hydrogel, ~ 2.12 for COA2 hydrogel, and ~ 1.90 for curcumin-loaded COA2 hydrogel) were considerably greater than that of the saline-treated group (~ 0.78), representing the low foreign body reaction and good biocompatibility of these hydrogels as brain implants in vivo.

**Fig. 9:**
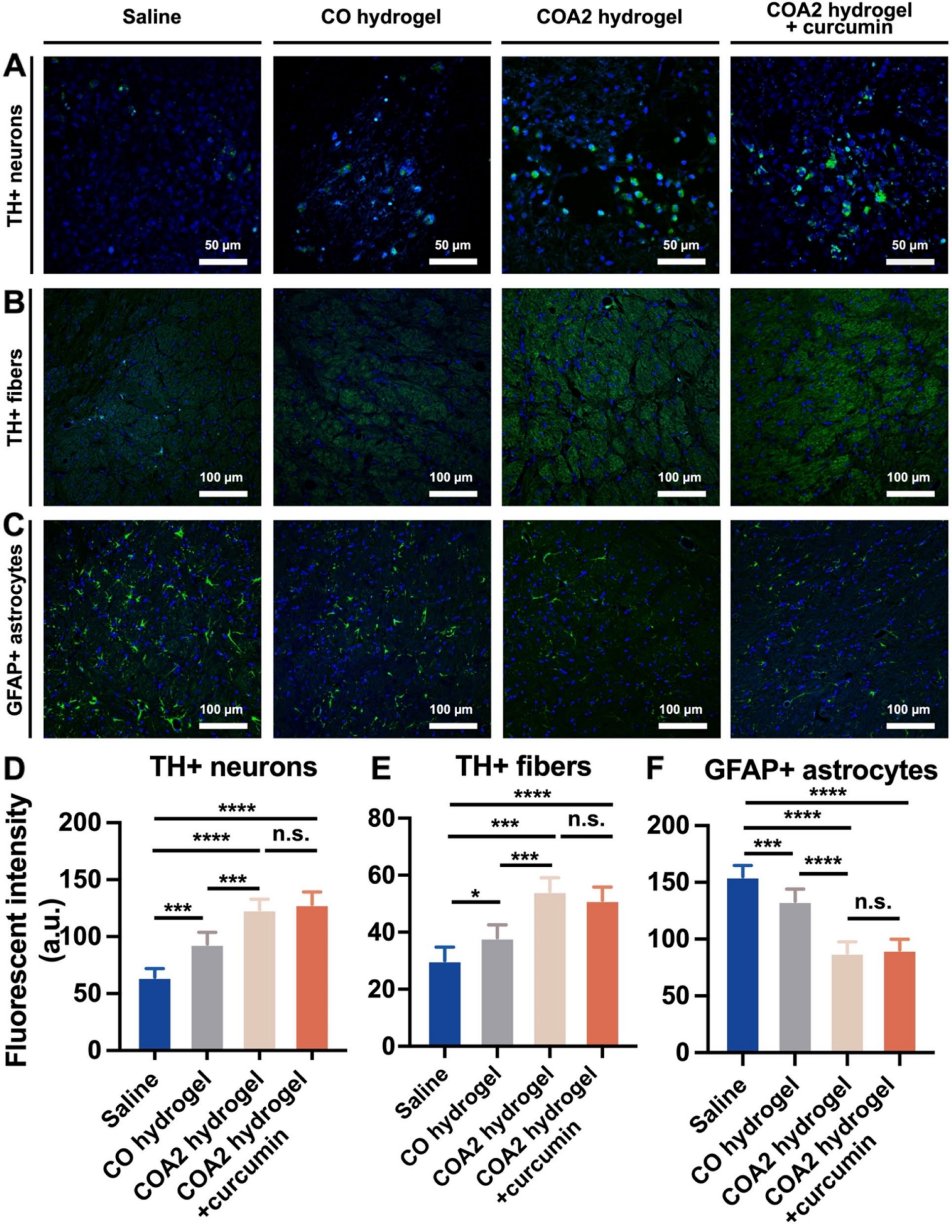
In vivo immunofluorescent analyses of each animal group for the related markers. The expression of (**A**) tyrosine hydroxylase positive (TH+) dopaminergic neurons in the substantia nigra pars compacta (SNpc), (**B**) TH+ dopaminergic fibers in the striatum, and (**C**) glial fibrillary acidic protein positive (GFAP+) astrocytes in the PD rat striatum were investigated at 14 days after surgery. Green color represents the indicated marker and blue color represents the nucleus. The fluorescent intensities were quantified and presented as graphics for (**D**) TH+ dopaminergic neurons in the SNpc, (**E**) TH+ dopaminergic fibers in the striatum, and (**F**) GFAP+ astrocytes in the striatum of each group. *p < 0.05, ***p < 0.001, and ****p < 0.0001 between the indicated groups

Histological staining demonstrated the presence of other factors associated with PD in sections of brain tissue. The expression of TH, one of the speed-limiting enzymes in the biosynthesis of dopamine, was examined in both SNpc and projected striatum on day 14 following implantation. The fluorescent images of TH positive (TH+) neurons in SNpc and TH+ fibers in the striatum are shown in Fig. 9A and B, respectively, with semi-quantitative data summarized in Fig. 9D and E. Compared with the saline group after 14 days, the hydrogel-treated PD rats showed significant increases in the number and fluorescence intensity of TH+ neurons in the SNpc, especially for COA2 and curcumin-loaded COA2 groups. The same tendency was observed in the fluorescence density of TH+ dopaminergic fibers at the striatum. Meanwhile, COA2 hydrogel and curcumin-loaded COA2 hydrogel groups did not show statistically significant difference in TH+ expression at 14 days. Another marker for the astrocyte response, GFAP, was also immunostained (Fig. 9C). The semi-quantitative data in Fig. 9F revealed that, as the course of PD progressed, there were significantly more GFAP positive (GFAP+) astrocytes in the saline-treated group vs. the other groups. The COA2 hydrogel and curcumin-loaded COA2 hydrogel groups exhibited lower numbers of GFAP+ astrocytes, indicating a reduction in astrocyte response by the COA2 hydrogel. Taken together, these in vivo findings supported the use of the bioactive COA2 hydrogel alone to treat 6-OHDA-induced PD. The COA2 hydrogel may reduce the loss of dopaminergic neurons and fibers in the SNpc and striatum while reducing the astrocyte response of PD rats.

## Discussion

Regeneration of a natural tissue-like microenvironment by implanting an engineered hydrogel that mimics the extracellular matrix requires consideration of structural support, force transfer, and the ability to regulate cell adhesion, proliferation, migration, and differentiation, etc. [38]. Designing a hydrogel that closely resembles the host tissue is a challenging task because of the need for functionality and bioactivity [39]. Also, the presence of various bioactive ingredients or properties in the hydrogel composition can conform the designed material to specific tissues, such as conductive hydrogels for neural tissues [20]. Conductive hydrogels are an emerging new generation of biomaterials for treating neurodegenerative diseases such as PD, particularly serving as cell/drug carriers during the treatment [40, 41]. In concord with the contemporary ways to treat PD, an implantable biomaterial for PD treatment should not only be biocompatible but also possess antioxidant and anti-inflammatory to neural tissues [42]. Meanwhile, the potential of using a conductive hydrogel alone was primarily validated in relieving behavioral abnormalities of PD rats [24]. Therefore, seeking a bioactive hydrogel with conductivity and strong antioxidant properties to treat PD and to compare the efficacy with the other antioxidant agent is critical to the development of novel therapeutic strategies for neurodegenerative diseases. In the present study, a bioactive conductive hydrogel was designed with proper physico-chemical properties as well as compelling antioxidant and anti-inflammatory capabilities for neuroprotection.

AuNPs are often used as additives in the preparation of conductive composite hydrogels [20]. Most studies have used negatively charged citrate-covered AuNPs, but their high hydrophobicity has proven to activate the innate immune system including TNF-α secretion [43–45]. The modification of AuNPs with some hydrophilic polymers, such as polyethylene glycol and TA, has been shown to confer some inflammation-regulating effects on modified AuNPs [46]. Meanwhile, tannins are water-soluble phenolic derivatives that are naturally synthesized and accumulated in higher plants. As the simplest and predominantly hydrolysable from of tannins, TA owns antioxidant, anti-inflammatory, and antiviral properties [47, 48]. The polyphenolic nature of TA, i.e., hydrophobic core and hydrophilic shell, allows TA to interact with cell surface proteins and present bioactivity [49]. The quinone-functionalized OTA can serve as a crosslinker but is not efficient [26]. To combine the characteristics of Au and TA as well as to generate a more effective Schiff crosslinker, the quinone-functionalized OTA@Au nano-crosslinker was synthesized in the present study by a two-step process. As mentioned, the synthesis of OTA was synthesized via laccase oxidization to generate quinone in situ by oxidation of accessible tyrosyl grafted phenol units [26]. Such reaction preferentially oxidized the phenolic hydroxyl group of the TA structure to become reactive o-quinones, which was validated in literature [50]. TA as a stabilizer to prepare TA modified AuNPs was also explored [25]. Inspired by the literature, the first effort in our current study was to optimize the reaction steps in order to synthesize a more effective OTA@Au nano-crosslinker successfully. IR spectroscopy verified that TA was oxidized by laccase to become OTA and have quinone groups. Based on the DLS measurement, a stable suspension of OTA@Au in aqueous medium was obtained. OTA is relatively stable in the low energy state after oxidation, so preparing OTA@Au nanoparticles as a reductant and stabilizer would not further oxidize OTA in the process, due to insufficient reaction conditions, e.g., the absence of a catalyst, in the system. The higher carbonyl content of OTA@Au nanocrosslinker than pristine OTA may support the hypothesis that the number of effective carbonyl groups in the pristine OTA was reduced due to self-aggregation, but the carbonyl groups in OTA@Au nanoparticles may be better exposed and result in more effective crosslinking via Schiff reaction. The morphology of OTA@Au was analyzed by coherent SAXS and examined by TEM, which indicated a spherical shape for the OTA@Au nano-crosslinker. Moreover, the OTA@Au retained its original spherical shape after mixing with CMC to prepare the conductive COA hydrogel.

The key for optimization of COA hydrogel is the content of OTA@Au nano-crosslinker that can be varied. First of all, the unconcentrated OTA@Au solution (100 ppm) was mixed with CMC solution (4 wt%). No macroscopic gel formation was observed in the mixture after 24 h. Therefore, both the OTA@Au nano-crosslinker dispersion and the CMC solution were tested in various concentrations in order to obtain an optimal composition for the final hydrogel. As the proportion of the CMC (main chain) in the hydrogel increased (2.5 wt%), the shape of the hydrogel was maintained for longer periods of time and the gelation time was shorter. Meanwhile, a concentration of OTA@Au surpassing 200 ppm (COA2) did not significantly enhance the chemical crosslinking as well as the conductivity further. Both COA1 hydrogel and COA2 hydrogel possessed the conductivity over 1.3 mS/cm, which falls in the appropriate range for neural tissue regeneration [51]. Besides, the crosslinking in COA1 hydrogel with a high concentration of OTA@Au (250 ppm in hydrogel) is too fast, which results in an unevenly mixed hydrogel. No proper way can resolve this problem unless decreasing the concentration of the crosslinker, i.e., as in COA2 hydrogel. The properties of such inhomogeneous COA1 cannot be precisely defined. Among the three hydrogels, COA2 hydrogel has a modulus greater than COA3 but the modulus is still lower than 200 Pa, which is similar to the modulus of native brain tissue and thus can promote neuronal differentiation. COA3 hydrogel has a long gelation time (~ 45 min) and degrades rather quickly under the physiological environment because of low crosslinking density. These properties are not favorable for the performance of the implanted hydrogel in a prolonged period. For the above-mentioned reasons, COA2 hydrogel with the composition CMC 2.5 wt%/OTA@Au 200 ppm was selected for major experiments. The crosslinking network of the COA hydrogel is primarily composed of the dynamic Schiff base linkages between quinone groups of OTA@Au and amine groups of CMC, and strengthened by non-covalent interactions such as hydrogen bonds [52]. The synthesis of OTA@Au nano-crosslinker on the basis of OTA showed improvement in crosslinking efficiency possibly owing to their nanoscale morphology as well as good dispersion that may facilitate the formation of Schiff base linkages and hydrogen bonds. The tiny 34G needle delivery, nearly complete self-healing, and self-adaptation properties, together with the favorable shear thinning behavior, signify the potential of COA2 hydrogel for the minimally invasive treatment. As for the PD rat model, these features enable administration of low-volume hydrogel into the brain through stereotactic techniques and allow the hydrogel to stay at the target site for Schiff bonding to the surrounding tissue and for self-adapting to prevent needle tracking.

NSCs exhibited improved proliferation and differentiation in COA2 versus CO hydrogels. The results from these in vitro studies indicated that COA2 hydrogel offered NSCs with permissive microenvironments to promote their growth. The cell proliferation rate constantly increased in two hydrogels through the 14 days of culture, especially in COA2 hydrogel. A chitosan-based electroconductive hydrogel with the ability to foster proliferation and differentiation of NSCs was recently reported, but such hydrogels failed to induce the neuronal-specific protein expression in NSCs [24]. NSCs encapsulated in COA2 hydrogels exhibited greater expression levels of glial- and neuronal-related genes than those in CO hydrogels after 14 days. Besides, the protein expression of the mature neuronal marker (MAP2) in COA2 hydrogel was significantly higher than that in CO hydrogel. Chitosan and TA are known to encourage the cell viability and neuronal-like cell differentiation of NSCs due to the presence of primary amines and chitooligosaccharides (degradation products of chitosan) [53, 54]. The better promotion of COA2 hydrogel versus CO hydrogel is thus contributed by OTA@Au nano-crosslinkers, in accordance with the literature finding that the conductivity conferred by AuNPs in a hydrogel is beneficial for the viability and differentiation of electroactive stem cells [21].

PD is associated with various mechanisms including neuroinflammation and oxidative stress, in which oxidative stress stimulates a feedforward cycle of neuronal cell inflammation and death, resulting in the production of ROS to drive disease progression [55]. Anti-inflammatory and antioxidative drugs have been described as promising treatments for PD [56]. A hydrogel intended for effective PD treatments should therefore possess antioxidative capabilities and anti-inflammatory properties. The COA2 hydrogel in this study rescued about 90% of the ROS-triggered inflamed NSCs after 12 h of encapsulation, better than a previously reported chitosan-based electroconductive hydrogel that rescued ~ 80% inflamed NSCs [24]. COA2 hydrogel contacting J774A.1 macrophages induced anti-inflammatory rather than pro-inflammatory effect in the in vitro assay.

When injected in vivo, the mechanical properties of the hydrogel and its integrity with the surrounding tissue play a significant role in suppressing the immune response of the host tissue [57, 58]. According to the literature [57], the mechanical strength of brain tissue ranges from 0.1 kPa to 2 kPa. The COA hydrogel developed by us has a strength of 0.12–0.21 kPa, which is in accordance with the strength of the relevant sites. Also, the crosslinking mechanism of COA hydrogel is Schiff reaction, a dynamic reaction existing in biological tissues, so that after implantation, the hydrogel not only has similar modulus to the surrounding tissues, but also adheres to the surrounding tissues with bonding to ensure the integrity between the host tissues and the hydrogel. Microglia (Iba-1+) and astrocytes (GFAP+) can give signals of inflammation and tissue damage, but they do not reveal whether the hydrogel implant has an anti-inflammatory or neuroprotective effect unless the microglia/macrophage polarization is explored [59, 60]. By staining CD86 and CD163 proteins and calculating the M2/M1 ratio, it is clear whether the microglia/macrophages present in the target area have a pro- or anti-inflammatory effect in the tissue. Accordingly, both COA2 hydrogel and curcumin-loaded COA2 hydrogel showed good biocompatibility, i.e., M2/M1 ratio > 1.5, and presented less microglia as well as less polarized macrophages than CO hydrogel- and saline-treated groups. Based on in vitro and in vivo results, the OTA@Au crosslinked chitosan hydrogel possessed antioxidant and anti-inflammatory capacities with neuroprotective effect, which may support its therapeutic potential in PD.

The therapeutic efficacy of the hydrogel was evaluated by the PD rat model, where the hydrogel was injected into the SNpc and assessed after 14 days. The 6-OHDA-induced PD rat model is better than the classic N-methyl-4-phenyl-1,2,3,6-tetrahydropyridine-induced PD mouse model in translation as well as in the stability of behavioral symptoms. Also, the pathological process of the former model is more similar to the progressive evolution of human PD neurodegeneration for evaluation of neuroprotection. Nevertheless, the model is rarely employed to evaluate the therapeutic effect of a biomaterial implant due to the complexity of stereotaxic technique and secondary surgery [61, 62]. In the present study, COA2 hydrogel and curcumin-loaded COA2 hydrogel showed positive effects on motor function recovery, which was verified by two behavior tests. Meanwhile, the COA2 hydrogel loaded with curcumin did not display a greater effect. Compared with the chitosan-polyurethane-Au hydrogel previously reported [24], COA2 and curcumin-loaded COA2 hydrogels have slightly lower electroconductivity, but they are superior to the earlier hydrogel in motor function recovery (Fig. S7). The greater efficacy of COA2 hydrogel suggests that OTA@Au nano-crosslinker in the present study work better than colloidal AuNPs in the earlier study.

Excessive STN burst discharges, a pathological electrophysiologic finding in PD, are causally related to parkinsonian locomotor impairments rather than just being an associated phenomenon [63]. In our study, the spike rates of STN discharges in the four experimental groups after treating for 14 days were obviously higher than that of healthy rats (~ 15 spikes/s) reported in the literature [64]. The electrophysiological signals in the COA2 hydrogel and curcumin-loaded COA2 hydrogel groups after 14 days were similar to that in PD rats before the secondary surgery, indicating an effective relief of the irregular discharge in STN and a prevention for further PD development. Since motor cortex is a target of dopaminergic innervation from SNpc [65], the implanted bioactive hydrogel in our study may have relieved PD symptoms by affecting neuronal cell rhythms in the STN of the SNpc projection area and reducing the deranged response of STN to motor cortex.

Nigrostriatal pathway involved in PD progression can connect the striatum to the SNpc, which is known as a target brain region for dopaminergic projections [66]. Injection of COA2 or curcumin-loaded COA2 hydrogel into the SNpc may recover motor function by protecting dopaminergic TH+ neurons and TH+ fibers in the SNpc and striatum. According to literature, GFAP+ astrocytes in excess can generate an acidic inflammatory environment that promotes microglia formation and continues neuroinflammation to exacerbate PD, while a moderate amount of astrocytes may release neuroprotective growth factors [41, 67]. In the PD rat model, both COA2 hydrogel and curcumin-loaded COA2 hydrogel groups demonstrated the reasonably lower amount of astrocytes and relatively more TH+ neurons and fibers than the other groups. The histological results indicated that these two groups exhibited good neuroprotective effects, which are consistent with the behavioral and electrophysiological findings. The curcumin-loaded COA2 hydrogel did not show significantly better therapeutic efficacy than the plain COA2 hydrogel probably because the antioxidative mechanism of curcumin overlays that of the bioactive COA2 hydrogel. Curcumin has been shown to exert anti-neurodegenerative effects by activating the inhibited hippocampal nerve regeneration-related signaling pathways in PD, but the onset time is at least 1 month [68, 69]. Additionally, curcumin as a natural antioxidant is functionally active due to the phenolic groups [70, 71], while OTA@Au nano-crosslinkers in COA hydrogel may generate potential bonding with curcumin to hinder or delay the treatment effects of curcumin. The therapeutic efficacy of COA2 hydrogel is related to its appropriate conductivity and modulus together with the distinctive anti-inflammatory and neuroprotective properties of the hydrogel. The bioactive and electroconductive hydrogel based on OTA@Au nano-crosslinkers thus has a strong potential to treat neurodegenerative diseases. The effect of such hydrogel can be as beneficial as a drug and may not only serve as a drug/cell carrier. Particularly, the prompt efficacy of the bioactive hydrogel may be comparable or complementary to an antioxidant and compensate for the time required before other antioxidant treatments work in diseases.

The possible mechanisms of COA hydrogel for PD treatment are summarized below. First, COA hydrogel can promote the proliferation and differentiation of NSCs toward neurons, as supported by the in vitro assay. The conductive COA hydrogel can regulate the irregular discharge behavior of neurons in the projection area of rat brain, as demonstrated by in vivo electrophysiology. From the view point of bioactive functions, COA hydrogel has the antioxidant and anti-inflammatory functions as well as the ability to rescue the inflamed cells, confirmed by in vitro experiments. Based on the histological evidence, COA hydrogel can decrease the generation of microglia to slow down the degeneration of TH+ neurons and nerve fibers as well as to reduce the accumulation of GFAP+ astrocytes with no significant foreign substance response. The positive therapeutic efficacy may be attributed to the capacity of COA hydrogel in removing the ROS generated by inflammatory glial cells, and to the bioactivity in rescuing inflammatory neuronal cells on the verge of degeneration to improve their function together with discharging behavior, thus slowing down the original vicious cycle of PD.

## Conclusion

New OTA-stabilized AuNPs with quinone groups as efficient nano-crosslinkers were synthesized and characterized. Bioactive self-healing hydrogels made of CMC and OTA@Au had proper modulus (120–210 Pa) and conductivity (1–1.4 mS/cm). The hydrogels (COA hydrogels) exhibited good injectability through tiny 34G needle (with 80 μm inner diameter), rapid self-healing (~ 100%), and shear-thinning properties. NSCs showed better cell proliferation in COA2 hydrogel (modulus ~ 180 Pa) versus non-Au-containing CO hydrogel, and inclined to differentiate towards neurons expressing the specific marker protein. The hydrogel was antioxidative and anti-inflammatory and rescued ~ 90% of inflamed NSCs in vitro. The biocompatibility and therapeutic efficacy of the injectable hydrogel as brain implants were confirmed in vivo by the PD rat model. The COA2 hydrogel implant effectively alleviated the irregular discharge of nerve cells in the intracerebral projection area, promoted the recovery of motor function, and reduced the histological neurodegeneration in PD rats, which was as effective as the drug-loaded hydrogel. These findings support the use of bioactive and conductive COA hydrogel alone as a promising biomaterial implant for neuroprotection and PD therapy instead of merely a cell/drug carrier.

## Supplementary Information

**Additional file 1: Table S1.** The primer sequences used for RT-PCR analyses of mouse NSCs. **Table S2.** The primer sequences used for RT-PCR analyses of J774A.1 macrophages. **Table S3.** The zeta potential and hydrodynamic diameter values of CMC and OTA@Au. **Table S4.** Chemical compositions, abbreviated names, and gelation time (time required for sol-to-gel transition) of the COA hydrogels prepared with different formulae. **Table S5.** Quantitative data from SEM images of the hydrogels (cross-sectional view). **p < 0.01 between the indicated groups. **Table S6.** The proliferation rates (%) of NSCs encapsulated in the CMC-based conductive hydrogel crosslinked with dialdehyde polyurethane containing nanogold as the positive control. **Fig. S1.** The TEM image for carboxymethyl chitosan (CMC). CMC was observed after negative staining of the CMC solution using phosphotungstic acid. **Fig. S2.** Macroscopic images of the hydrogels. **Fig. S3.** The SEM image for the cross-section of the CO hydrogel. **Fig. S4.** The rheological data by strain sweep experiments of (A) CO hydrogel and (B) COA2 hydrogel in the range of 0.1 to 800% dynamic strain amplitudes at 1 Hz frequency. Orange arrows showed the gel-to-sol points and the corresponding strain values. **Fig. S5.** SAXS profiles for each single raw materials, including CMC and OTA@Au. **Fig. S6.** Photos for the animal experiments, including (A) 6-OHDA neurotoxin lesion, (B) the spontaneous circling speed test (red arrow: circling direction), (C) the cylinder asymmetry test (red arrow: forelimb contact), and (D) the electrophysiological experiments of the PD rats. **Fig. S7.** Comparison of the efficacy based on behavioral (A) circling speed evaluation and (B) cylinder asymmetry evaluation after treatment for 14 days between the present hydrogels and the optimized hydrogel in previous literature (CDAH2 hydrogel) [24]. **p < 0.01, ***p < 0.001, and ****p < 0.0001 between the indicated groups. **Fig. S8.** In vivo immunohistochemical analyses of Iba-1 positive microglia (brown) for the explanted tissue after implantation in the brain for 14 days. Cell nuclei were stained in blue color.

## Data Availability

The supplementary data are available online at the website of BioMed Central. The datasets used and/or analyzed during the current study are available from the corresponding author on reasonable request.

## Abbreviations

PD: Parkinson’s disease
NSCs: Neural stem cells
STN: Subthalamic nucleus
ROS: Reactive oxygen species
CMC: Carboxymethyl chitosan
TA: Tannic acid
AuNPs: Gold nanoparticles
OTA: Oxidized tannic acid
OTA@Au: Oxidized tannic acid modified gold nanoparticles
TEM: Transmission electron microscopy
UV-Vis: Ultraviolet-visible
COA: Carboxymethyl chitosan/oxidized tannic acid modified gold nanoparticles
SEM: Scanning electron microscope
DI: Deionized
PBS: Phosphate buffered saline
G’: Storage modulus
G”: Loss modulus
SAXS: Small-angle X-ray scattering
q: Scattering vector
HG-DMEM: High-glucose Dulbecco’s modified Eagle’s medium
FBS: Fetal bovine serum
G418: Geneticin
PSA: Penicillin–streptomycin–amphotericin
CCK-8: Cell Counting Kit-8
RT-PCR: Real-time reverse transcriptase–polymerase chain reaction
GFAP: Glial fibrillary acidic protein
MAP2: Microtubule-associated protein 2
GAPDH: 3-phosphoglycerate dehydrogenase
PFA: Paraformaldehyde
BSA: Bovine serum albumin
IFN-γ: Interferon gamma
IL-1β: Interlukin-1β
IL-6: Interlukin-6
IL-10: Interlukin-10
TNP-α: Tumor necrosis factor
6-OHDA: 6-hydroxydopamine
SNpc: Substantia nigra pars compacta
AP: Anterior-posterior
LAT: Lateral
DEP: Depth
TH: Tyrosine hydroxylase
