# Supplementary material for: Bioactive self-healing hydrogel based on tannic acid modified gold nano-crosslinker as an injectable brain implant for treating Parkinson’s disease
Source: Biomater Res. 2023 Feb 8;27:8. doi: 10.1186/s40824-023-00347-0 (PMC9909866; doi:10.1186/s40824-023-00347-0)
Supplement: Supplementary file 1 — Additional file 1: Table S1. The primer sequences used for RT-PCR analyses of mouse NSCs. Table S2. The primer sequences used for RT-PCR analyses of J774A.1 macrophages. Table S3. The zeta potential and hydrodynamic diameter values of CMC and OTA@Au. Table S4. Chemical compositions, abbreviated names, and gelation time (time required for sol-to-gel transition) of the COA hydrogels prepared with different formulae. Table S5. Quantitative data from SEM images of the hydrogels (cross-sectional view). **p < 0.01 between the indicated groups. Table S6. The proliferation rates (%) of NSCs encapsulated in the CMC-based conductive hydrogel crosslinked with dialdehyde polyurethane containing nanogold as the positive control. Fig. S1. The TEM image for carboxymethyl chitosan (CMC). CMC was observed after negative staining of the CMC solution using phosphotungstic acid. Fig. S2. Macroscopic images of the hydrogels. Fig. S3. The SEM image for the cross-section of the CO hydrogel. Fig. S4. The rheological data by strain sweep experiments of (A) CO hydrogel and (B) COA2 hydrogel in the range of 0.1 to 800% dynamic strain amplitudes at 1 Hz frequency. Orange arrows showed the gel-to-sol points and the corresponding strain values. Fig. S5. SAXS profiles for each single raw materials, including CMC and OTA@Au. Fig. S6. Photos for the animal experiments, including (A) 6-OHDA neurotoxin lesion, (B) the spontaneous circling speed test (red arrow: circling direction), (C) the cylinder asymmetry test (red arrow: forelimb contact), and (D) the electrophysiological experiments of the PD rats. Fig. S7. Comparison of the efficacy based on behavioral (A) circling speed evaluation and (B) cylinder asymmetry evaluation after treatment for 14 days between the present hydrogels and the optimized hydrogel in previous literature (CDAH2 hydrogel) [24]. **p < 0.01, ***p < 0.001, and ****p < 0.0001 between the indicated groups. Fig. S8. In vivo immunohistochemical analyses of Iba-1 positive microglia [file 40824_2023_347_MOESM1_ESM.docx]

**Supporting information**

**Bioactive self-healing hydrogel based on tannic acid modified gold nano-crosslinker as an injectable brain implant for treating Parkinson’s disease**

Junpeng Xu^1^, Tsai-Yu Chen^1^, Chun-Hwei Tai^2,*^, Shan-hui Hsu^1,3,*^

^1^ Institute of Polymer Science and Engineering, National Taiwan University, No. 1, Sec. 4 Roosevelt Road, Taipei, Taiwan, Republic of China

^2^ Department of Neurology, National Taiwan University Hospital, No.7, Zhongshan S. Rd., Zhongzheng Dist., Taipei, Taiwan, Republic of China

^3^ Institute of Cellular and System Medicine, National Health Research Institutes, Miaoli, Taiwan, Republic of China

**Pages: 10**

**Supplemental Tables: 6**

**Supplemental Figures: 8**

*** Corresponding author:**

**Shan-hui Hsu (ORCID: 0000-0003-3399-055X)**

Email: shhsu@ntu.edu.tw; Tel.: +886-2-3366-5313; Fax: +886-2-3366-5237

**Chun-Hwei Tai (ORCID: 0000-0002-3493-8879)**

Email: chtai1502@ntu.edu.tw; Tel.: +886-2-2313-3456 ext.65336; Fax: +886-2-2341-8395

**Table S1.** The primer sequences used for RT-PCR analyses of mouse NSCs.

| **Gene** | **Primer** | |
| --- | --- | --- |
|  | **Forward** | **Reverse** |
| mGADPH | GGCTACAGCAACAGGGTGGT | CGAGTTGGGATAGGGCCTCT |
| mNestin | ACTGTGGAATCACCAGGAGG | ATTCCACCTCTCCCAGAGAC |
| mGFAP | CTGAACCCTCTGAGCAAATG | GAATCAAACACAGAGCCTGC |
| mβ-Tubulin | CAGGGCCAAGACAAGCAGCA | GGAGCCCTAATGAGCTGGTGA |
| mMAP2 | TTCTCCACTGTGGCTGTTTG | GAGCCTGTTTGTAGACTGGAAGA |

**Table S2.** The primer sequences used for RT-PCR analyses of J774A.1 macrophages.

| **Gene** | **Primer** | |
| --- | --- | --- |
|  | **Forward** | **Reverse** |
| mβ-actin | TCCTGTGGCATCCACGAAACT | GGAGCAATGATCCTGATCTTC |
| mIL-1 | CCCAAGCAATACCCAAAGAAGAAG | TGTCCTGACCACTGTTGTTTCC |
| mIL-6 | TTCCATCCAGTTGCCTTCTTG | TCATTTCCACGATTTCCCAGAG |
| mIL-10 | CACAAAGCAGCCTTGCAGAA | CTGGCCCCTGCTGATCCT |
| mTNF-α | CGAGTGACAAGCCTGTAGCC | TTGAAGAGAACCTGGGAGTAGAC |

**Table S3.** The zeta potential and hydrodynamic diameter values of CMC and OTA@Au.

| **Sample** | **Zeta potential**  **(mV)** | **Hydrodynamic diameter (nm)** |
| --- | --- | --- |
| CMC | -38.3 ± 5.4 |  |
| OTA@Au | -45.6 ± 2.7 | 30.26 ± 1.79 |

**Table S4.** Chemical compositions, abbreviated names, and gelation time (time required for sol-to-gel transition) of the COA hydrogels prepared with different formulae.

| **Abbreviated**  **name** | **CMC**  **(wt%)** | **OTA@Au**  **(ppm)** | **OTA**  **(ppm)** | **Gelation time (min)** |
| --- | --- | --- | --- | --- |
| CO | 2.5 |  | 200 | ~25 |
| COA1 | 2.5 | 250 |  | ~6 |
| COA2 | 2.5 | 200 |  | ~15 |
| COA3 | 2.5 | 150 |  | ~45 |
| COA4 | 2 | 250 |  | ~60 |
| COA5 | 2 | 200 |  | ~120 |
| COA6 | 2 | 150 |  | ~360 |

**Table S5.** Quantitative data from SEM images of the hydrogels (cross-sectional view). ***p* < 0.01 between the indicated groups.

|  | **CO hydrogel** | **COA2 hydrogel** | **Statistical**  **analysis** |
| --- | --- | --- | --- |
| **Pore size (diameter) / μm** | 76.12 ± 9.30 | 59.72 ± 6.70 | ** |
| **Wall thickness / μm** | 2.01 ± 0.25 | 1.59 ± 0.27 | ** |

**Table S6.** The proliferation rates (%) of NSCs encapsulated in the CMC-based conductive hydrogel crosslinked with dialdehyde polyurethane containing nanogold as the positive control.

| **Time (days)** | 0 | 2 | 4 |
| --- | --- | --- | --- |
| **Proliferation rate** | 100.0 ± 9.1 | 116.0 ± 8.0 | 222.1 ± 11.3 |
| **Time (days)** | 6 | 10 | 14 |
| **Proliferation rate** | 326.5 ± 31.5 | 1481.1 ± 22.6 | 629.0 ± 47.6 |

**Fig. S1.** The TEM image for carboxymethyl chitosan (CMC). CMC was observed after negative staining of the CMC solution using phosphotungstic acid.

**Fig. S2.** Macroscopic images of the hydrogels.

**
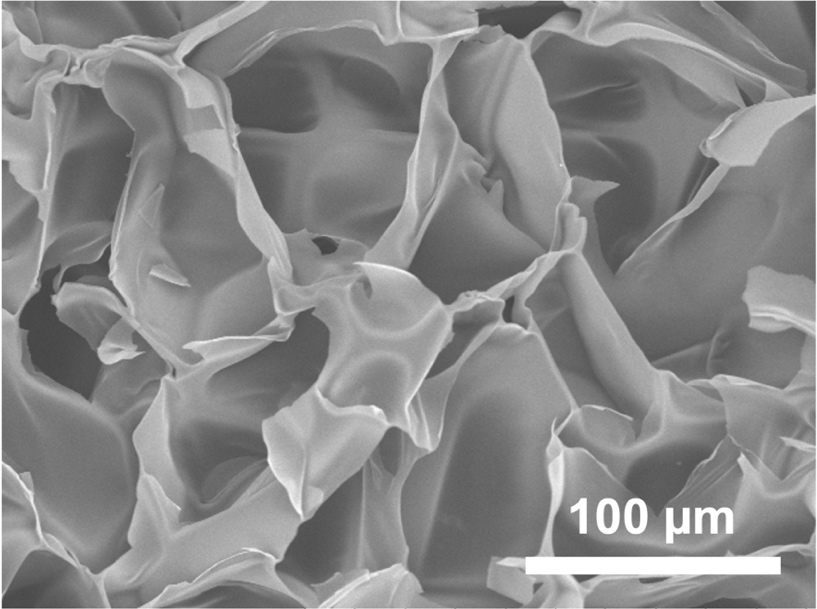
Fig. S3.** The SEM image for the cross-section of the CO hydrogel.


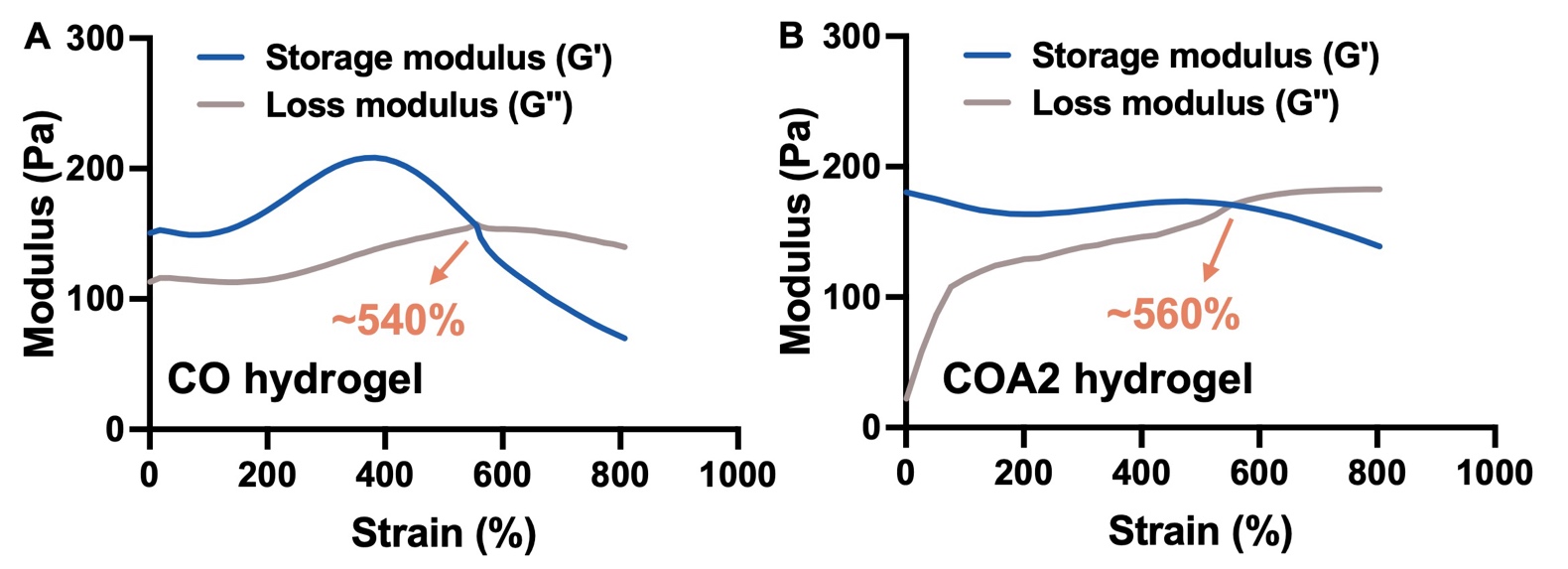


**Fig. S4.** The rheological data by strain sweep experiments of (A) CO hydrogel and (B) COA2 hydrogel in the range of 0.1% to 800% dynamic strain amplitudes at 1 Hz frequency. Orange arrows showed the gel-to-sol points and the corresponding strain values.


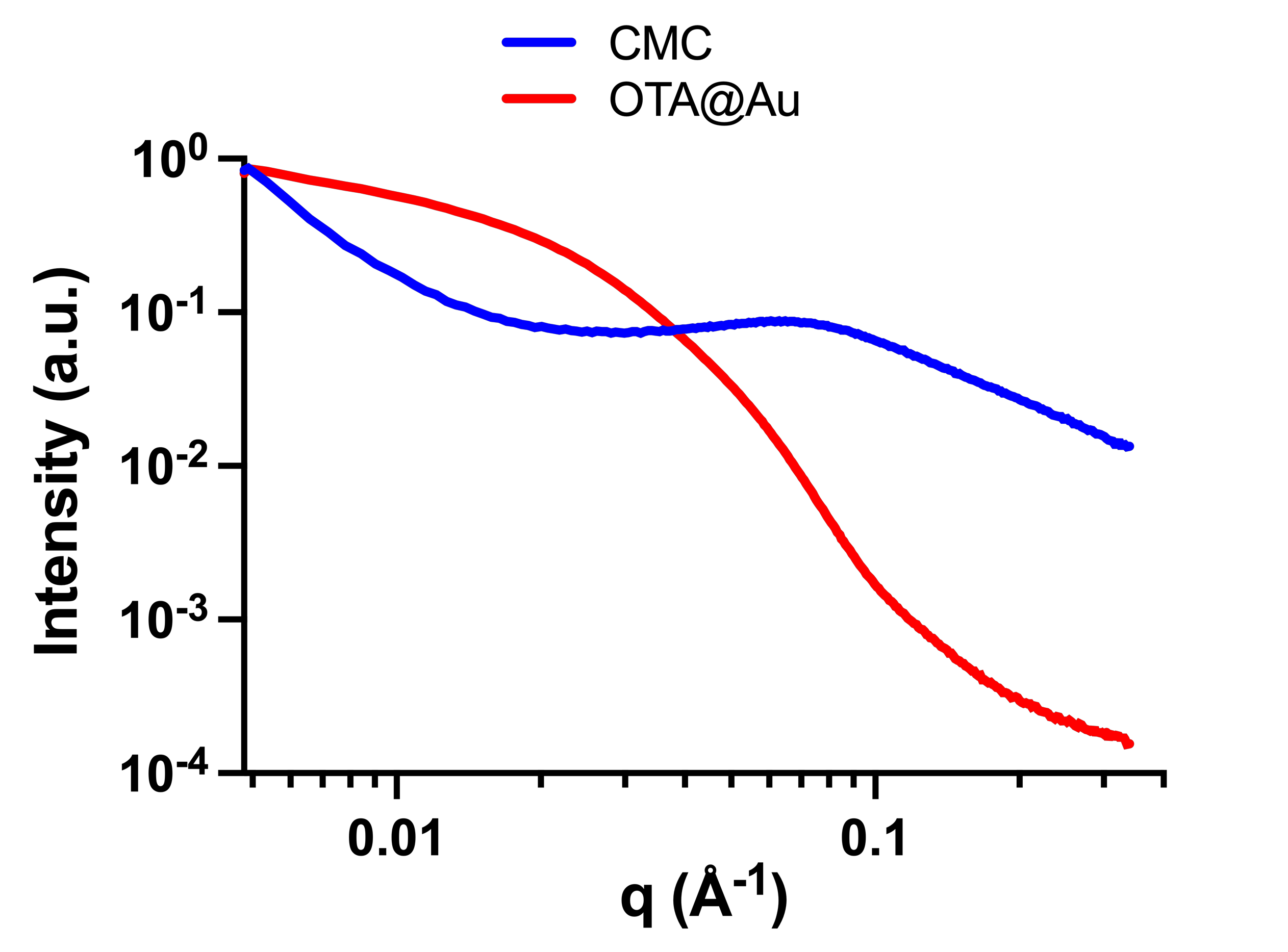


**Fig. S5.** SAXS profiles for each single raw materials, including CMC and OTA@Au.


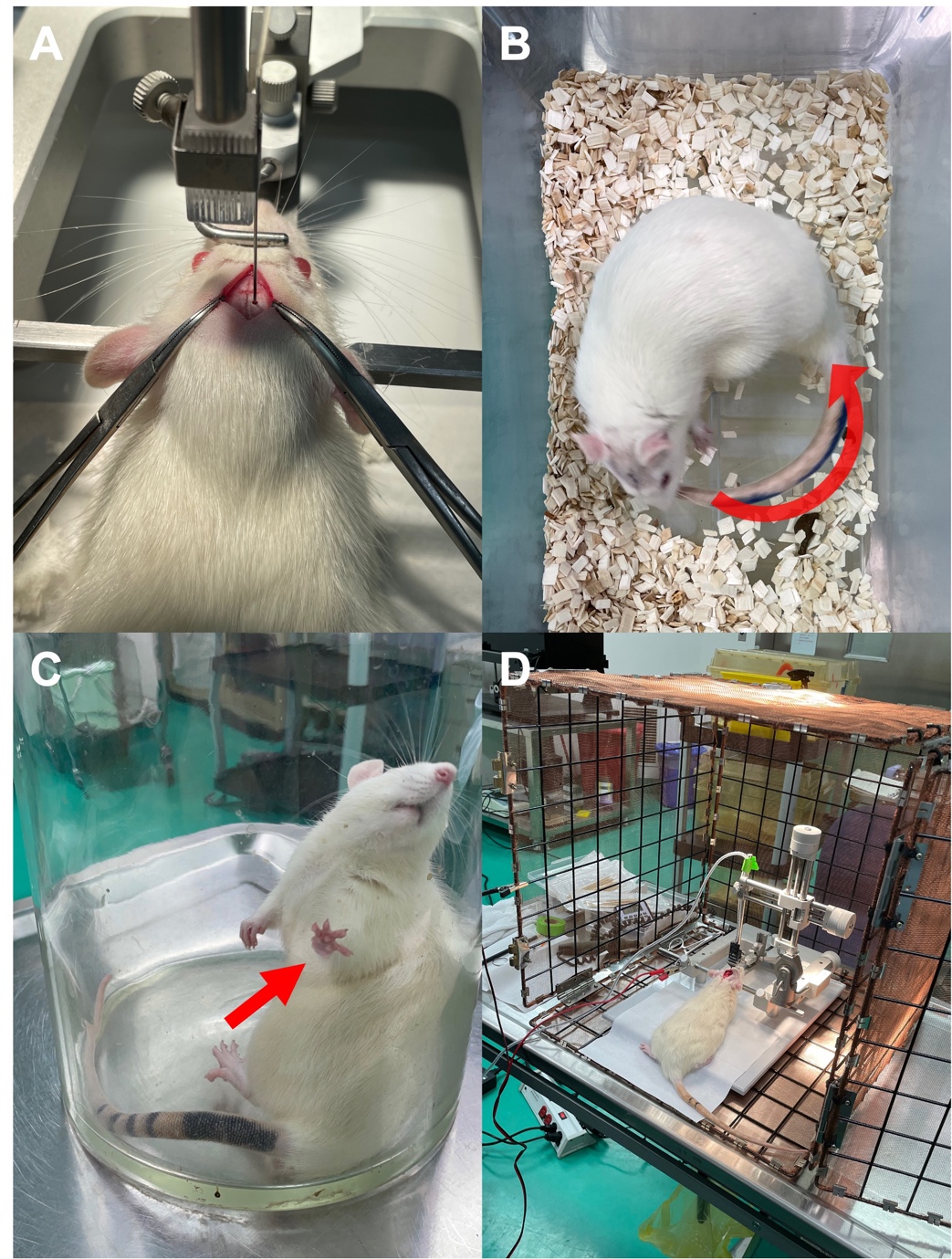


**Fig. S6.** Photos for the animal experiments, including (A) 6-OHDA neurotoxin lesion, (B) the spontaneous circling speed test (red arrow: circling direction), (C) the cylinder asymmetry test (red arrow: forelimb contact), and (D) the electrophysiological experiments of the PD rats.


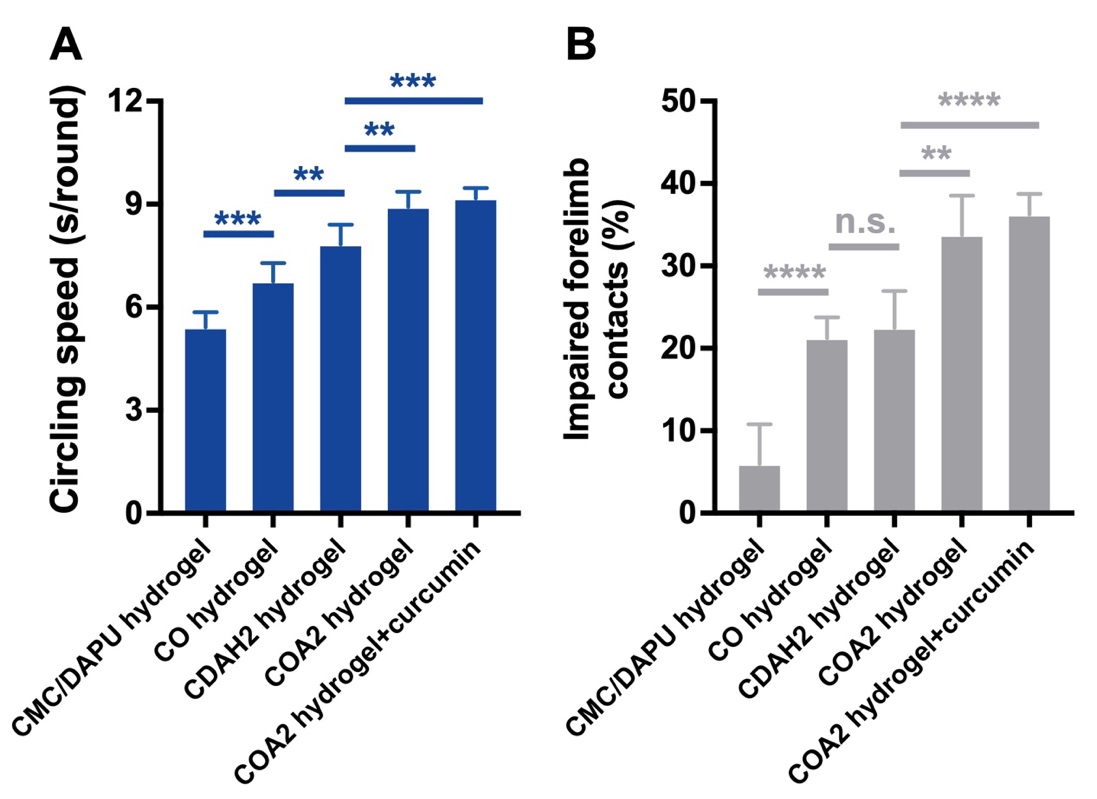


**Fig. S7.** Comparison of the efficacy based on behavioral (A) circling speed evaluation and (B) cylinder asymmetry evaluation after treatment for 14 days between the present hydrogels and the optimized hydrogel in previous literature (CDAH2 hydrogel) [1]. ***p* < 0.01, ****p* < 0.001, and *****p* < 0.0001 between the indicated groups.

**Fig. S8.** *In vivo* immunohistochemical analyses of Iba-1 positive microglia (brown) for the explanted tissue after implantation in the brain for 14 days. Cell nuclei were stained in blue color.

**Reference:**

[1] J. Xu, C.-H. Tai, T.-Y. Chen, S.-h. Hsu, An anti-inflammatory electroconductive hydrogel with self-healing property for the treatment of Parkinson’s disease, Chemical Engineering Journal 2022; 446:137180. <https://doi.org/https://doi.org/10.1016/j.cej.2022.137180>.
